# SpaDiT: diffusion transformer for spatial gene expression prediction using scRNA-seq

**DOI:** 10.1093/bib/bbae571

**Published:** 2024-11-07

**Authors:** Xiaoyu Li, Fangfang Zhu, Wenwen Min

**Affiliations:** School of Information Science and Engineering, Yunnan University, 650500, Kunming, Yunnan, China; School of Health and Nursing, Yunnan Open University, 650599, Kunming, China; School of Information Science and Engineering, Yunnan University, 650500, Kunming, Yunnan, China

**Keywords:** diffusion model, spatial transcriptomics data, scRNA-seq data, transformer, gene expression prediction

## Abstract

The rapid development of spatially resolved transcriptomics (SRT) technologies has provided unprecedented opportunities for exploring the structure of specific organs or tissues. However, these techniques (such as image-based SRT) can achieve single-cell resolution, but can only capture the expression levels of tens to hundreds of genes. Such spatial transcriptomics (ST) data, carrying a large number of undetected genes, have limited its application value. To address the challenge, we develop SpaDiT, a deep learning framework for spatial reconstruction and gene expression prediction using scRNA-seq data. SpaDiT employs scRNA-seq data as an a priori condition and utilizes shared genes between ST and scRNA-seq data as latent representations to construct inputs, thereby facilitating the accurate prediction of gene expression in ST data. SpaDiT enhances the accuracy of spatial gene expression predictions over a variety of spatial transcriptomics datasets. We have demonstrated the effectiveness of SpaDiT by conducting extensive experiments on both seq-based and image-based ST data. We compared SpaDiT with eight highly effective baseline methods and found that our proposed method achieved an 8%–12% improvement in performance across multiple metrics. Source code and all datasets used in this paper are available at https://github.com/wenwenmin/SpaDiT and https://zenodo.org/records/12792074.

## Introduction

Single-cell RNA sequencing (scRNA-seq) can represent the entire transcriptome of a specific cell in an organ, providing an excellent perspective for in-depth study of various behaviors and mechanisms between cells [1]. However, since scRNA-seq must undergo sample tissue dissociation, it also leads to the inability of scRNA-seq to capture the spatial distribution and spatial information of cells, which is often crucial for understanding the complex physiological processes between cells [2]. Therefore, spatial transcriptomics (ST) has emerged as an advanced technology that can retain spatial location information while measuring gene expression in tissue or cell samples [3]. This technology enables researchers to parse the spatial distribution of gene expression in tissues, enhancing the understanding of cell types, functions, interactions, and key details in development, disease, and biological processes [4].

At present, ST technology can be mainly divided into two categories: based on next-generation sequencing technology (seq-based): such as 10x Visium [5], Slide-seq [6], and Stereo-seq [7], transcriptome-wide gene expression within a spatial point can be detected. Fluorescence in situ hybridization (image-based), such as seqFish [8] and MERFISH [9], can measure thousands of genes at the resolution of single cells, but they usually lack full transcriptome coverage, resulting in only a few hundred genes in actual sequencing. Although these two technologies can detect gene expression in the whole transcriptome range, their capture rate is low due to their resolution [10, 11]. The current solution mainly focuses on increasing the capture rate and predicting uncaptured genes by using scRNA-seq data to enhance ST data to improve its resolution [12–14].

In recent years, a variety of methods have been proposed to use scRNA-seq data to improve the resolution of ST data and predict uncaptured genes. These methods are Tangram [15], scVI [16], SpaGE [17], stplus [18], SpaOTsc [19], novoSpaRc [20], SpatialScope [21], and stDiff [22]. They all assume that scRNA-seq data and ST data have similar expression distributions, and they identify the similarity between scRNA-seq cells and ST cells by detecting the expression patterns of shared genes. Then, these methods use similar scRNA-seq cells to predict the unmeasured part of ST data [23]. However, due to the sparse nature of scRNA-seq and ST data, and the reliance on common genes to calculate similarity, this poses a huge challenge to how to align the two data. In addition, simply using scRNA-seq as a reference for ST data prediction is difficult to avoid introducing batch bias of scRNA, which increases the difficulty of predicting unknown genes [24].

In this paper, we introduce a novel method named SpaDiT, which uses a conditional diffusion model to understand and generate unmeasured gene expression in ST data. Although diffusion models have made significant contributions in the field of computer vision and have shown excellent performance in the field of protein or drug generation [25–28], their application in genomics is still relatively limited. The goal of SpaDiT is to utilize scRNA-seq as a prior input in the diffusion model to help the model understand the relationship between gene expressions, thereby guiding the model to generate genes that are not measured in ST data. SpaDiT utilizes genes in single cells as unique identifiers by incorporating them in the diffusion model along with the corresponding genes in ST, and employs the Transformer-based diffusion model to enhance the model’s prediction accuracy of specific genes.

We conduct a comprehensive performance evaluation on 10 ST datasets based on different sequencing technologies, different tissues, and different sample sizes, and compare them with the current state of the art (SOTA) methods. The results show that our model achieves the best performance on all five evaluation indicators, and the correlation between predicted gene expression and actual gene expression shows the best accuracy. This shows that SpaDiT can effectively make predictions when predicting unmeasured gene expression in ST data. In addition, the genes predicted by our model have a high spatial similarity with the genes in the actual ST data. For the spatial expression patterns of each data set, our model can accurately predict and clearly divide the spatial boundaries. This demonstrates SpaDiT’s ability to predict ST data gene expression and provide subsequent analysis.

## Materials and methods

### Datasets and pre-processing

In this paper, we collected ten benchmark datasets (scRNA sequencing and spatial transcriptomics data) from different tissues of various organisms. As illustrated in Table 1, these datasets originate from various biological organizations and utilize differing sequencing platforms and technologies. They also vary in sample sizes, number of spatially measured genes, and missing data rates. Specifically, the sequencing platforms for single-cell data in these datasets include 10X Chromium, Smart-seq, and Smart-seq2. For spatial transcriptomics data, the platforms are seqFISH, MERFISH, 10X Visium, STARmap, and Slide-seqV2. These datasets are derived from different biological tissues, primarily from mouse and human breast cancer tissue sections.

**Table 1:** The list of ten paired scRNA-seq and spatial transcriptomic datasets with corresponding tissue types, sequencing platforms, and preprocessing information. The first five ST datasets are image-based, while the next five are sequencing-based. **Prepro. Cells/Spots** and **Prepro. Genes** columns show the number of cells/spots and genes remaining after preprocessing. **Dropout Rate** represents the proportion of missing values (zeros) in the data. HPR: hypothalamic preoptic region; PMC: primary motor cortex.

			**Platform**		**Number of Cells/Spots**		**Number of Genes**		**Prepro. Cells/Spots**		**Prepro. Genes**		**Dropout Rate**	
**Datasets**	**Tissue**	**GEO ID**	SC	ST	SC (Cells)	ST (Spots)	SC (Genes)	ST (Genes)	SC (Cells)	ST (Spots)	SC (Genes)	ST (Genes)	SC	ST
MH [29]	mouse hippocampus	GSE158450	10X Chromium	seqFish	8596	3585	16384	249	8584	3585	1260	249	80.3%	6.3%
MHPR [9]	mouse HPR	GSE113576	10X Chromium	MERFISH	31299	4975	18646	154	31297	4975	1939	153	73.7%	62.2%
ML [30]	mouse liver	GSE109774	Smart-seq2	seqFISH	981	2177	17533	19532	887	2177	2279	569	73.2%	75.4%
MG [30]	mouse gastrulation	GSE15677	10X Chromium	seqFISH	4651	8425	19103	351	4651	8425	1945	345	58.6%	74.1%
MVC [31]	mouse visual cortex	-	Smart-seq	STARmap	14249	1549	34041	1020	14249	1549	3774	844	58.2%	76.2%
MHM [32]	mouse hindlimb muscle	GSE161318	10X Chromium	10X Visium	4816	995	15460	33217	4809	995	1667	416	80.3%	68.9%
HBC [33]	human breast cancer	CID3586	10X Chromium	10X Visium	6178	4784	21164	28402	6143	4784	625	125	76.6%	70.6%
ME [34]	mouse embryo	GSE160137	10X Chromium	10X Visium	3415	198	19374	53574	3415	198	2163	540	61.1%	62.3%
MPMC [35]	mouse PMC	-	10X Chromium	10X Visium	3499	9852	24340	24518	3499	9852	2544	636	70.6%	81.7%
MC [36]	mouse cerebellum	SCP948	10X Chromium	Slide-seqV2	26252	41674	24409	23264	26252	41674	822	205	79.5%	83.9%

We preprocessed each dataset with the following steps: (1) Removal of low-quality cells. For scRNA-seq datasets, cells expressing less than 500 genes were filtered out. For spatial transcriptomics datasets, spots expressing less than 1 gene were filtered out. (2) Normalization of the expression matrix. For spatial transcriptomics datasets, to normalize the expression matrices, we used the following equation:


(1) \begin{align*}& D_{ij} = \log \left( \frac{N \times C_{ij}}{\sum_{j} C_{ij}} + 1 \right),\end{align*}

where $ C_{ij} $ represents the raw read count for gene $ j $ in spot $ i $, $ D_{ij} $ represents the normalized read count for gene $ j $ in spot $ i $, and $ N $ is the median number of detected transcripts per cell. For scRNA-seq datasets, we normalized their expression matrix using the function NormalizeData and default parameters in Seurat 3.2. (3) Selection of highly variable genes is crucial for subsequent analysis. These genes, characterized by their high variability, were selected for further study. The program identified the top 25% of genes with the highest expression variability, which typically exhibit greater biological significance and more accurately represent inter-sample differences. The coefficient of variation for each gene was calculated using the following equation:


(2) \begin{align*}& CV_{i} = \frac{\sigma_{i}}{\mu_{i}},\end{align*}

where $ CV_{i} $ is the coefficient of variation of gene $ i $, $ \sigma _{i} $ is the s.d. of the spatial distribution of gene $ i $ in all spots, and $ \mu _{i} $ is the average expression of gene $ i $ in all spots. We partitioned the processed data into training, validation, and test sets with ratios of 7:2:1, respectively. These subsets are mutually independent, with the test set being strictly separate from the training set. All reported results were derived solely from evaluations on the test set. All genes in ST were almost completely retained after re-processing. In our study, the term “samples” refers to genes, specifically those sequenced in scRNA and spatial transcriptomics data.

The high dropout rates in spatial transcriptomics data can be attributed to several factors. First, the limited sensitivity of current spatial transcriptomics technologies often leads to undetected low-expressing genes, causing dropout events. Additionally, certain cell types have lower RNA content, which increases the probability of dropouts. Tissue heterogeneity, with variations in cell density and RNA concentration across regions, further contributes to dropout rates. Finally, biases in sample preparation, amplification, and sequencing disproportionately affect low-abundance transcripts.

In this study, the validation set primarily serves the purpose of hyperparameter tuning. Specifically, the validation set is utilized to monitor model performance during training, assess generalization ability through changes in four indicators, and adjust hyperparameters to prevent overfitting. Additionally, the validation set aids in selecting between different models or model versions, ensuring that the final chosen model performs optimally on this set. All experimental results in this paper are obtained on the test set, and all test sets have never appeared in the training set.

### The architeture of SpaDiT

**Figure 1:**
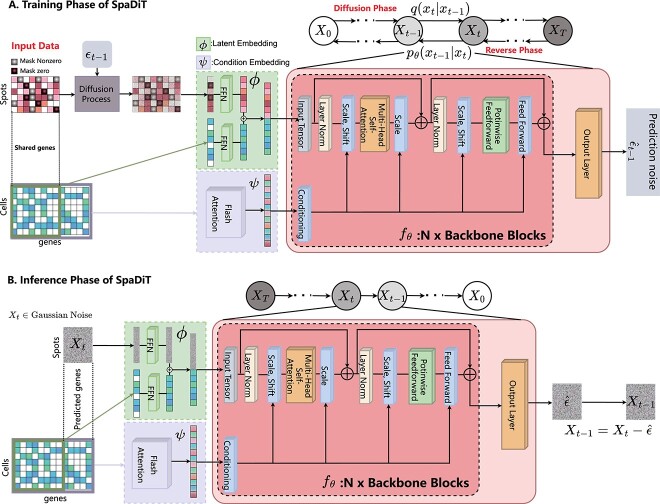
The architecture of SpaDiT. There are three parts in total: latent embedding, conditional embedding, and network backbone. (A) is the training process where each gene is considered as a sample, and (B) is the inference process.

SpaDiT is a conditional diffusion-based deep generative model that enhances spatial transcriptomics data by leveraging single-cell RNA sequencing (scRNA-seq) data as prior information, aiming to accurately predict the expression of unmeasured or unknown genes. As illustrated in the Fig. 1, SpaDiT takes two types of input data: a gene expression matrix from spatial transcriptomics data and another from scRNA-seq data. Utilizing a conditional diffusion model, SpaDiT uses scRNA-seq data as a conditioning factor to guide the model through the diffusion and denoising processes, thereby generating the targeted gene expression profiles for the spatial transcriptomic data. The SpaDiT architecture comprises three key modules: the Latent Embedding module for processing spatial transcriptomic data, the Condition Embedding module for processing scRNA-seq data, and the core network architecture: Diffusion with Transformer, which facilitates the integration and generation of data. In the following sections, we will introduce the main modules of SpaDiT.

#### Latent Embedding in SpaDiT

In the proposed SpaDiT, the latent embedding module is crucial. Instead of operating directly on real data, we work within an efficient, low-dimensional latent space, which is better suited for likelihood-based generative models. Therefore, we utilize an encoder to map the high-dimensional input data to a low-dimensional representation, and we train the diffusion model within this latent space.

Notably, our proposed method involves two types of data input: spatial transcriptomics data ($X_{\text{st}}$) and scRNA-seq data ($X_{\text{sc}}$). The genes in $X_{\text{sc}}$ are divided into shared genes ($G_{\text{share}}$) with spatial transcriptomics data and unique genes ($G_{\text{unique}}$). For the input of Latent Embedding, we define it as follows: for each sample (i.e. gene) $ x_{\text{st}}^{i} \in X_{\text{st}} $ in the spatial transcription data, we integrate it with the scRNA-seq data $ x_{\text{sc}}^{i} \in X_{\text{sc}} $, where $ x_{\text{st}}^{i} $ and $ x_{\text{sc}}^{i} $ in $ G_{\text{share}} $ are utilized as inputs. In latent embedding, we employ a simple feed-forward network to project $ x_{\text{st}}^{i} $ and $ x_{\text{sc}}^{i} $ into the same dimensional space, concatenating the projected $ \hat{x}_{\text{st}}^{i} $ and $ \hat{x}_{\text{sc}}^{i} $ as the output of the latent embedding, that is, $ x_\phi = \hat{x}_{\text{st}}^{i} \oplus \hat{x}_{\text{sc}}^{i} $.

#### Condition Embedding in SpaDiT

The condition embedding module leverages scRNA-seq data as a conditioning factor in our model, integrating it into the diffusion process to guide the model in generating the required gene expression. Given that scRNA-seq data is high-dimensional, directly using the entire matrix as input would result in the curse of dimensionality. Consequently, the condition embedding module utilizes an attention mechanism to convert the high-dimensional single-cell data matrix into a lower-dimensional representation. This reduces the data to a low-dimensional, high-expression latent representation, which is then used as a conditional mechanism in subsequent diffusion model training.

For the input of Condition Embedding, the high-dimensional input matrix $ X_{\text{sc}} $ is processed using Flash-attention to compute a lower-dimensional representation $ X_{\psi } $ as output:


(3) \begin{align*}& \begin{aligned} Q &= X_{\text{sc}} W^{Q}, K = X_{\text{sc}} W^{K}, V = X_{\text{sc}} W^{V}, \\ X_{\psi} &= \text{softmax}\left(\frac{Q \Phi_{K}(K)^{T}}{\sqrt{d_{k}}}\right) \Phi_{V}(V) \end{aligned}\end{align*}

where:

-
$ W^{Q} $ , $ W^{K} $, and $ W^{V} $ are projection matrices that transform $ X $ into queries $ Q $, keys $ K $, and values $ V $, respectively.
-
$ \Phi _{K} $ and $ \Phi _{V} $ are the dimensionality reduction functions applied to $ K $ and $ V $, resulting in lower-dimensional.
-
$ d_{k} $ is the dimension of $ K $ after projection, used to scale the softmax computation.
- The $\text{softmax}$ function is applied over each row, normalizing the dot product scores into a probability distribution used to compute the weighted sum of values $ \Phi _{V}(V) $.

#### Diffusion with Transformer in SpaDiT

The backbone network of our proposed SpaDiT is Diffusion Transformers (DiTs), a new architecture for diffusion models. For the backbone network model, we refer to previous work [37] and make modifications based on the challenges we encounter. Our backbone model has two types of input: $x_\phi $, representing latent embedding, and $x_\psi $, representing condition embedding. We initialize each residual block in the backbone network as an identity function and incorporate the condition embedding into the backbone. At each layer, we also perform scaling regression on all residual connections within the backbone, facilitating rapid model convergence.

After the final DiT block, the gene expression token sequence needs to be decoded into output noise prediction and output diagonal covariance prediction. The shapes of both outputs are identical to the input in the original space, and a standard linear decoder is employed to achieve this. Finally, the decoded tokens are rearranged to match the layout of the original expression, yielding the predicted noise and covariance.

#### Training phase in SpaDiT

Here in, SpaDiT works with two types of input data: the spatial transcriptomics data $X_{\text{st}} = \{ x_{\text{st}}^{i} \}_{i}^{n} \in \mathbb{R}^ {n \times p}$ and scRNA-seq data $X{\text{sc}} = \{ x_{\text{sc}}^{j} \}_{j}^{m} \in \mathbb{R}^ {m \times q}$. Among them, $n$ and $p$ respectively represent the number of genes and the number of spots in spatial transcriptomics data, and $m$ and $q$ respectively represent the number of genes and the number of cells in scRNA-seq data.

The training phase of SpaDiT is shown in the Fig. 1 (A). We first mask the genes of the original spatial transcriptomics data according to a certain proportion, where the mask is divided into two parts: the part with an expression value of zero and the part with an expression value that is not zero. The input tensor of the training phase is defined as follows:


(4) \begin{align*}& x_{0} = \hat{x}_{\text{st}}^{i} \oplus \hat{x}_{\text{sc}}^{i},\end{align*}

where $ \hat{x}_{\text{st}}^{i} $ and $ \hat{x}_{\text{sc}}^{i} $ are projection of $ x_{\text{st}}^{i} $ and $ x_{\text{sc}}^{i} $ by a simple feed-forward network.

In the realm of DDPM (Denoising Diffusion Probabilistic Models) [25, 38], consider the task of learning a model distribution $ p_\theta (\mathbf{x}_{0}) $ that closely approximates a given data distribution $ q(\mathbf{x}_{0}) $. Suppose we have a sequence of latent variables $ \mathbf{x}_{t} $ for $ t = 1, \ldots , T $, existing within the same sample space as $ \mathbf{x}_{0} $, which is denoted as $ \mathcal{X} $. DDPMs (Denoising Diffusion Probabilistic Models) are latent variable models that are composed of two primary processes: the forward process and the reverse process. The forward process is defined by a Markov chain, as follows:


(5) \begin{align*}& q(\mathbf{x}_{1:T} | \mathbf{x}_{0}):= \prod_{t=1}^{T} q(\mathbf{x}_{t} | \mathbf{x}_{t-1}),\end{align*}

where $q(\mathbf{x}_{t} | \mathbf{x}_{t-1}):= \mathcal{N}(\sqrt{1 - \beta _{t}}\mathbf{x}_{t-1}$, and the variable $\beta _{t}$ is a small positive constant indicative of a noise level. The sampling of $x_{t}$ can be described by the closed-form expression $q(x_{t} | x_{0}) = \mathcal{N}(x_{t}; \sqrt{\alpha _{t}}x_{0}, (1 - \alpha _{t})\mathbf{I})$, where $\hat{\alpha }_{t}:= 1 - \beta _{t}$ and $\alpha _{t}$ is the cumulative product $\alpha _{t}:= \prod _{i=1}^{t} \hat{\alpha }_{i}$. Consequently, $x_{t}$ is given by the equation $x_{t} = \sqrt{\alpha _{t}}x_{0} + (1 - \alpha _{t})\epsilon $, with $\epsilon \sim \mathcal{N}(0, \mathbf{I})$. In contrast, the reverse process aims to denoise $x_{t}$ to retrieve $x_{0}$, a process that is characterized by the ensuing Markov chain:


(6) \begin{align*}& \begin{aligned} &p_\theta(\mathbf{x}_{0:T}):= p(\mathbf{x}_{T}) \prod_{t=1}^{T} p_\theta(\mathbf{x}_{t-1} | \mathbf{x}_{t}), \quad \mathbf{x}_{T} \sim \mathcal{N}(0, \mathbf{I}), \\ &p_\theta(\mathbf{x}_{t-1} | \mathbf{x}_{t}):= \mathcal{N}(\mathbf{x}_{t-1}; \mu_\theta(\mathbf{x}_{t}, t), \sigma^{2}_\theta(\mathbf{x}_{t}, t)\mathbf{I}), \\ & \mu_\theta(\mathbf{x}_{t}, t) = \frac{1}{\alpha_{t}} \left( \mathbf{x}_{t} - \frac{\beta_{t}}{\sqrt{1-\alpha_{t}}} \epsilon_\theta(\mathbf{x}_{t}, t) \right), \\ & \sigma_\theta(\mathbf{x}_{t}, t) = \beta_{t}^{1/2} \end{aligned}\end{align*}

where $\epsilon _\theta (\mathbf{x}_{t}, t)$ is a trainable denoising function and


(7) \begin{align*}& \beta_{t} = \begin{cases} \frac{1-\bar{\alpha}_{t-1}}{1-\bar{\alpha}_{t}} \beta_{1}, & \text{for} t> 1, \\ \beta_{1}, & \text{for} t = 1. \end{cases}\end{align*}

SpaDiT aims to help models understand and estimate the expression of missing genes in ST data by utilizing scRNA-seq data as prior information, thereby enabling the model to better predict gene expression from ST data. We represent the data of the condition as $x_{0}^{c} = x_{\psi }$. Therefore, our goal is to estimate the posterior $p((E_{n,1} - m_{1}) \odot ((E_{n,1} - m_{2}) \odot x_{0}))| x_{0}^{c})$, where $E_{n, 1}$ is an all-1 matrix $n\times 1$ with dimension, $m_{1}, m_{2} \in \{ 0, 1 \}^{n \times 1} $ is an element-wise indicator, representing the zero and non-zero parts of the mask respectively.

We also denote predicted genes as $x_{t}^{*}$, where t is the time step. Therefore, the goal of our SpaDiT conditional mechanism is to estimate the probability:


(8) \begin{align*}& p_\theta(x_{t-1}^{*} | x_{t}^{*}, x_{0}^{c}).\end{align*}

In order to better use the scRNA-seq data as a priori conditions for the diffusion model to perform predicting gene expression, we transform the Equation (5) and Equation (6) into:


(9) \begin{align*}& \begin{split} &p_\theta(x_{0:T}^{*} | x_{ 0}^{c}):= p(x_{T}^{*}) \prod_{t=1}^{T} p_\theta(x_{t-1}^{*} | x_{ t}^{*}, x_{ 0}^{c}), x_{ T}^{*} \sim \mathcal{N}(0, \mathbf{I}).\\ &p_\theta(x_{t-1}^{*} | x_{t}^{*}, x_{ 0}^{c}):= \mathcal{N}(x_{ t-1}^{*}; \mu_\theta(x_{ t}^{*}, t | x_{0}^{c}), \sigma_\theta(x_{t}^{*}, t | x_{0}^{c})\mathbf{I}). \end{split}\end{align*}

We can optimize the Equation (9) parameters by minimizing the variational lower bound:


(10) \begin{align*}& \mathbb{E}_{q}\left[ -\log p_{\theta}(x_{0} \mid x_{ 0}^{c}) \right] \leq \mathbb{E}_{q}\left[ -\log \frac{p_{\theta}(x_{ 0:T} \mid x_{0}^{c})}{q(x_{ 1:T} \mid x_{0})} \right].\end{align*}

Also we can get a simplified training objective:


(11) \begin{align*}& \mathbb{E}_{x_{0}\sim q(\mathbf{x}_{0}), \epsilon\sim\mathcal{N}(0,\mathbf{I}),t}\|(\epsilon - \epsilon_{\theta}(\mathbf{x}_{t}^{*}, t | x_{ 0}^{c}))\|^{2}_{2}.\end{align*}

#### Inference phase in SpaDiT

We focus on improving the conditional diffusion model characterized by the inverse process described in Equation (9). Our goal is to accurately model the conditional distribution $p\left (x_{t-1}^{*} | x_{t}^{*}, x_{ 0}^{c}\right )$ without resorting to approximations. To achieve this, we adapt the parameterization of DDPM from Equation (6) for the conditional setting. We introduce a conditional denoising function $\epsilon _{\theta }: \left (\mathcal{X}^{*} \times \mathbb{R} \mid \mathcal{X}^{c}\right ) \rightarrow \mathcal{X }^ *$ accepts conditional observation value $x_{ 0}^{c}$ as input parameter. On this basis, we use $\epsilon _{\theta }$ for parameterization, as follows:


(12) \begin{align*}& \begin{split} \mu_{\theta}(x_{t}^{*},t | x_{ 0}^{c}) &= \mu \left(x_{ t}^{*}, t, s_{\theta}\left(x_{ t}^{*}, t | x_{ 0}^{c}\right)\right),\\ \sigma_{\theta}(x_{ t}^{*}, t | x_{ 0}^{c}) &= \sigma \left(x_{ t}^{*}, t\right), \end{split}\end{align*}

where $\mu $ and $\sigma $ are defined in Equation (6). Utilizing the function $\epsilon _{\theta }$ and the data $x_{0}$, we can simulate samples of $x_{ 0}^{*}$ by employing the reverse process outlined in Equation (9).

For more details on the diffusion model, please see the Supplementary Notes 1 to 6.

### Algorithm of SpaDiT

Our model employs the Diffusion Transformer (DiT) structure, with its network backbone rooted in the Transformer architecture. The SpaDiT structure is specifically crafted to seamlessly integrate conditional information into the generative model, guiding the diffusion step within the generation process. In SpaDiT, the conditional embedding is initially encoded via a Flash-Attention Encoder to adjust its dimensions for alignment with the Transformer’s input. This encoded conditional vector serves as an additional “key” and “value” input in each Transformer layer, integrating the conditional vector into the key (K) of the self-attention mechanism. This approach ensures that the attention score calculation reflects not only the internal relationships within the input data but also the relevance to the condition. This allows the model to concentrate on the most pertinent aspects of the input data under the guidance of specific conditions.

To more clearly explain how the conditional mechanism participates in the model training and inference process, we provide the algorithm pseudo code of the SpaDiT method. We provide the training procedure of SpaDiT in Algorithm 1 and the inference procedure of SpaDiT in Algorithm 2. For Implementation Details of SpaDiT, please see the Supplementary Note 7.




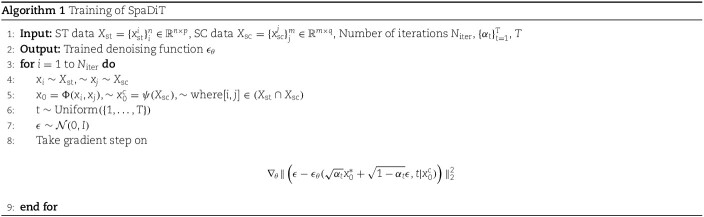






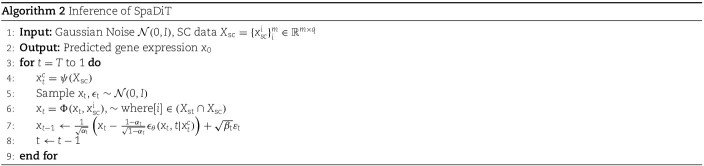



### Evaluation metrics

To evaluate the performance of SpaDiT and other baseline methods, we use five evaluation indicators: Pearson Correlation Coefficient (PCC), Structural Similarity Index Measure (SSIM), Root Mean Square Error (RMSE), Jensen–Shannon Divergence (JS), and Accuracy Score (AS) to evaluate the gene prediction performance of different methods on ten datasets. The specific definition of the evaluation metrics can be found in Supplementary Note 8.

### Baselines

We compared the performance of SpaDiT to eight baseline methods, with data processing procedures (e.g. normalization and scaling) consistent for each method. The specific baselines are as follows: Tangram [15], scVI [16], SpaGE [17], stPlus [18], SpaOTsc [19], novoSpaRc [20], SpatialScope [21], and stDiff [22]. A detailed introduction of each baseline can be found in the Supplementary Note 9.

## Results

### SpaDiT improves prediction accuracy of spatial gene expression

To rigorously assess the SpaDiT method’s capabilities in predicting gene expression, we conducted a comparative analysis with eight other widely recognized methods in the field. We used four key performance metrics, to systematically evaluate both SpaDiT and the comparator baseline methods. The evaluation focused on computing the mean and variance of these metrics across all genes within each dataset. The results are depicted in Table 2.

**Table 2:** Comparison with baseline methods on the ten paired scRNA-seq and ST datasets.

PCC$\uparrow $	MG	MH	MHPR	MVC	MHM	HBC	ME	MPMC	MC	ML
Tangram [15]	0.458$\pm $0.203	0.523$\pm $0.116	0.683$\pm $0.012	0.623$\pm $0.117	0.536$\pm $0.053	0.703$\pm $0.142	0.503$\pm $0.025	0.727$\pm $0.026	0.745$\pm $0.003	0.714$\pm $0.056
scVI [16]	0.476$\pm $0.157	0.446$\pm $0.157	0.691$\pm $0.143	0.594$\pm $0.023	0.511$\pm $0.117	0.656$\pm $0.005	0.496$\pm $0.007	0.716$\pm $0.014	0.736$\pm $0.015	0.637$\pm $0.001
SpaGE [17]	0.526$\pm $0.114	0.438$\pm $0.163	0.653$\pm $0.063	0.603$\pm $0.107	0.545$\pm $0.226	0.639$\pm $0.025	0.512$\pm $0.013	0.753$\pm $0.066	0.769$\pm $0.011	0.653$\pm $0.007
stPlus [18]	0.503$\pm $0.233	0.401$\pm $0.037	0.483$\pm $0.231	0.574$\pm $0.059	0.476$\pm $0.007	0.597$\pm $0.111	0.526$\pm $0.026	0.689$\pm $0.007	0.701$\pm $0.099	0.699$\pm $0.014
SpaOTsc [19]	0.522$\pm $0.014	0.485$\pm $0.107	0.657$\pm $0.002	0.629$\pm $0.147	0.496$\pm $0.018	0.587$\pm $0.107	0.547$\pm $0.006	0.734$\pm $0.201	0.738$\pm $0.064	0.723$\pm $0.005
novoSpaRc [20]	0.563$\pm $0.158	0.567$\pm $0.252	0.613$\pm $0.146	0.656$\pm $0.037	0.515$\pm $0.003	0.647$\pm $0.122	0.569$\pm $0.013	0.756$\pm $0.015	0.756$\pm $0.015	0.766$\pm $0.056
SpatialScope [21]	0.612$\pm $0.143	0.582$\pm $0.183	0.637$\pm $0.031	0.683$\pm $0.114	0.547$\pm $0.103	0.733$\pm $0.183	0.563$\pm $0.056	0.769$\pm $0.022	0.776$\pm $0.006	**0.803$\pm $0.014**
stDiff [22]	0.482$\pm $0.021	0.527$\pm $0.013	0.621$\pm $0.007	0.601$\pm $0.043	0.471$\pm $0.009	0.544$\pm $0.021	0.553$\pm $0.014	0.629$\pm $0.011	0.604$\pm $0.019	0.736$\pm $0.099
**SpaDiT (Ours)**	**0.657$\pm $0.035**	**0.621$\pm $0.099**	**0.770 $\pm $0.043**	**0.725$\pm $0.106**	**0.573$\pm $0.083**	**0.772$\pm $0.057**	**0.590$\pm $0.146**	**0.808$\pm $0.043**	**0.812$\pm $0.039**	0.784$\pm $0.096
SSIM$\uparrow $	MG	MH	MHPR	MVC	MHM	HBC	ME	MPMC	MC	ML
Tangram [15]	0.355$\pm $0.114	0.541$\pm $0.203	0.681$\pm $0.025	0.653$\pm $0.115	0.388$\pm $0.109	0.656$\pm $0.007	0.521$\pm $0.047	**0.889$\pm $0.043**	0.789$\pm $0.004	0.689$\pm $0.005
scVI [16]	0.487$\pm $0.155	0.422$\pm $0.128	0.647$\pm $0.121	0.564$\pm $0.025	0.374$\pm $0.115	0.617$\pm $0.028	0.587$\pm $0.013	0.674$\pm $0.012	0.736$\pm $0.006	0.694$\pm $0.014
SpaGE [17]	0.503$\pm $0.003	0.403$\pm $0.158	0.631$\pm $0.011	0.611$\pm $0.004	0.401$\pm $0.006	0.588$\pm $0.189	0.513$\pm $0.064	0.653$\pm $0.011	0.667$\pm $0.055	0.703$\pm $0.023
stPlus [18]	0.533$\pm $0.114	0.367$\pm $0.127	0.657$\pm $0.176	0.656$\pm $0.007	0.426$\pm $0.013	0.638$\pm $0.221	0.479$\pm $0.023	0.627$\pm $0.103	0.693$\pm $0.011	0.736$\pm $0.014
SpaOTsc [19]	0.547$\pm $0.126	0.503$\pm $0.013	0.701$\pm $0.026	0.637$\pm $0.021	0.484$\pm $0.170	0.626$\pm $0.118	0.601$\pm $0.188	0.663$\pm $0.114	0.718$\pm $0.004	0.688$\pm $0.007
novoSpaRc [20]	0.587$\pm $0.028	0.537$\pm $0.026	0.713$\pm $0.123	0.631$\pm $0.018	0.477$\pm $0.201	0.633$\pm $0.107	0.622$\pm $0.023	0.726$\pm $0.055	0.726$\pm $0.006	0.705$\pm $0.006
SpatialScope [21]	0.612$\pm $0.016	0.588$\pm $0.014	0.731$\pm $0.054	0.674$\pm $0.026	**0.512$\pm $0.122**	0.659$\pm $0.055	**0.701$\pm $0.022**	0.826$\pm $0.014	0.753$\pm $0.014	0.714$\pm $0.003
stDiff [22]	0.463$\pm $0.017	0.548$\pm $0.118	0.673$\pm $0.013	0.576$\pm $0.007	0.462$\pm $0.017	0.514$\pm $0.012	0.563$\pm $0.017	0.598$\pm $0.019	0.701$\pm $0.023	0.688$\pm $0.017
**SpaDiT (Ours)**	**0.632$\pm $0.037**	**0.574$\pm $0.125**	**0.738$\pm $0.044**	**0.689$\pm $0.114**	0.495$\pm $0.175	**0.717$\pm $0.111**	0.688$\pm $0.144	0.781$\pm $0.050	**0.787$\pm $0.042**	**0.751$\pm $0.107**
RMSE$\downarrow $	MG	MH	MHPR	MVC	MHM	HBC	ME	MPMC	MC	ML
Tangram [15]	1.263$\pm $0.053	1.412$\pm $0.018	1.263$\pm $0.012	1.587$\pm $0.041	1.237$\pm $0.005	1.542$\pm $0.003	1.633$\pm $0.004	1.324$\pm $0.048	1.216$\pm $0.184	1.346$\pm $0.015
scVI [16]	1.155$\pm $0.012	1.363$\pm $0.026	1.374$\pm $0.026	1.327$\pm $0.106	1.213$\pm $0.103	1.378$\pm $0.005	1.581$\pm $0.013	1.207$\pm $0.034	1.179$\pm $0.067	1.411$\pm $0.056
SpaGE [17]	1.187$\pm $0.025	1.433$\pm $0.037	1.287$\pm $0.029	1.354$\pm $0.047	1.347$\pm $0.025	1.413$\pm $0.101	1.553$\pm $0.024	1.137$\pm $0.011	1.213$\pm $0.005	1.233$\pm $0.008
stPlus [18]	1.254$\pm $0.003	1.367$\pm $0.045	1.384$\pm $0.121	1.289$\pm $0.022	1.156$\pm $0.014	1.331$\pm $0.077	1.496$\pm $0.033	1.656$\pm $0.007	1.154$\pm $0.024	1.303$\pm $0.014
SpaOTsc [19]	1.433$\pm $0.058	1.213$\pm $0.058	1.203$\pm $0.027	1.253$\pm $0.007	1.227$\pm $0.058	1.203$\pm $0.114	1.403$\pm $0.004	1.227$\pm $0.026	1.016$\pm $0.007	1.263$\pm $0.005
novoSpaRc [20]	1.275$\pm $0.143	1.526$\pm $0.213	1.252$\pm $0.011	1.206$\pm $0.014	1.412$\pm $0.117	1.198$\pm $0.007	1.556$\pm $0.021	1.334$\pm $0.015	0.967$\pm $0.153	1.523$\pm $0.007
SpatialScope [21]	1.019$\pm $0.022	1.288$\pm $0.258	1.201$\pm $0.003	**1.009$\pm $0.007**	1.217$\pm $0.005	1.102$\pm $0.005	1.483$\pm $0.007	1.104$\pm $0.056	**0.863$\pm $0.004**	1.343$\pm $0.014
stDiff [22]	1.326$\pm $0.019	1.325$\pm $0.022	1.081$\pm $0.013	1.219$\pm $0.066	1.312$\pm $0.007	1.217$\pm $0.023	1.561$\pm $0.023	1.326$\pm $0.016	1.224$\pm $0.003	1.223$\pm $0.009
**SpaDiT (Ours)**	**0.877$\pm $0.049**	**1.103$\pm $0.015**	**1.184$\pm $0.058**	1.116$\pm $0.038	**1.125$\pm $0.060**	**0.992$\pm $0.045**	**1.376$\pm $0.118**	**1.089$\pm $0.038**	1.004$\pm $0.037	**1.121$\pm $0.047**
JS$\downarrow $	MG	MH	MHPR	MVC	MHM	HBC	ME	MPMC	MC	ML
Tangram [15]	0.477$\pm $0.057	0.254$\pm $0.003	0.458$\pm $0.033	**0.343$\pm $0.007**	0.502$\pm $0.056	0.397$\pm $0.105	0.803$\pm $0.026	0.403$\pm $0.056	0.547$\pm $0.005	0.347$\pm $0.014
scVI [16]	0.426$\pm $0.088	0.324$\pm $0.147	0.496$\pm $0.011	0.403$\pm $0.001	0.537$\pm $0.113	0.427$\pm $0.089	0.749$\pm $0.015	0.423$\pm $0.115	0.601$\pm $0.014	0.363$\pm $0.047
SpaGE [17]	0.437$\pm $0.054	0.272$\pm $0.023	0.511$\pm $0.007	0.387$\pm $0.114	0.528$\pm $0.007	0.415$\pm $0.026	0.882$\pm $0.003	0.374$\pm $0.004	0.617$\pm $0.006	0.403$\pm $0.011
stPlus [18]	0.481$\pm $0.146	0.288$\pm $0.057	0.503$\pm $0.014	0.399$\pm $0.005	0.488$\pm $0.125	0.439$\pm $0.005	0.814$\pm $0.036	0.393$\pm $0.005	0.576$\pm $0.004	0.423$\pm $0.016
SpaOTsc [19]	0.513$\pm $0.126	0.334$\pm $0.058	0.411$\pm $0.022	0.403$\pm $0.147	0.503$\pm $0.111	0.411$\pm $0.015	0.792$\pm $0.007	0.417$\pm $0.011	0.463$\pm $0.026	**0.311$\pm $0.007**
novoSpaRc [20]	0.488$\pm $0.003	0.401$\pm $0.017	0.389$\pm $0.005	0.412$\pm $0.003	0.496$\pm $0.015	0.429$\pm $0.085	0.683$\pm $0.015	0.401$\pm $0.005	0.431$\pm $0.005	0.401$\pm $0.006
SpatialScope [21]	0.403$\pm $0.002	0.263$\pm $0.174	0.366$\pm $0.007	0.389$\pm $0.008	0.487$\pm $0.026	0.455$\pm $0.002	0.622$\pm $0.150	0.389$\pm $0.107	0.407$\pm $0.014	0.355$\pm $0.014
stDiff [22]	0.467$\pm $0.001	0.412$\pm $0.015	0.387$\pm $0.021	0.461$\pm $0.011	0.467$\pm $0.021	0.456$\pm $0.011	0.663$\pm $0.017	0.436$\pm $0.022	0.432$\pm $0.063	0.396$\pm $0.007
**SpaDiT (Ours)**	**0.346$\pm $0.012**	**0.246$\pm $0.005**	**0.337$\pm $0.010**	0.369$\pm $0.029	**0.463$\pm $0.116**	**0.381$\pm $0.061**	**0.549$\pm $0.134**	**0.356$\pm $0.012**	**0.371$\pm $0.013**	0.421$\pm $0.064

Our findings indicate that SpaDiT consistently achieves state-of-the-art (SOTA) performance in all four metrics across the ten examined datasets. However, it is important to note that in a few cases, SpaDiT slightly lags behind some established methods in one particular metric. This deviation provides critical insights into scenarios where SpaDiT might be further optimized.

**Figure 2:**
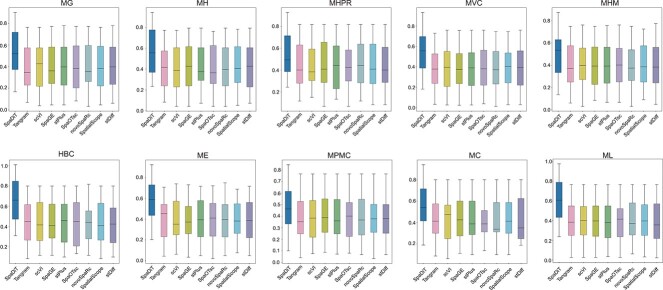
Performance evaluation is based on the comprehensive metric of Accuracy Score (AS) on ten real paired ST and scRNA-seq datasets. The figure shows the performance evaluation based on the comprehensive metric Accuracy Score (AS) across ten real paired spatial transcriptomics (ST) and single-cell RNA-seq (scRNA-seq) datasets. Accuracy Score is a composite indicator used to evaluate the overall performance of each model (Supplementary Note 1). In each boxplot, the central line represents the median, the box depicts the interquartile range (IQR), and the whiskers extend to 1.5 times the IQR. Individual points represent the AS values for each dataset. This figure provides a clear comparison of the performance of different integration methods across diverse datasets, reflecting both the stability and accuracy of the models.

In addition to these traditional metrics, we introduced an advanced scoring system, referred to as AS metrics, to further evaluate SpaDiT’s performance. The results, illustrated in Fig. 2, confirm that SpaDiT not only meets but often exceeds the performance benchmarks set by the baseline methods across all ten spatial transcriptomics (ST) datasets. The inclusion of AS metrics provides a more nuanced understanding of SpaDiT’s predictive prowess, underscoring its robustness and effectiveness in diverse experimental conditions. This comprehensive approach solidifies SpaDiT’s position as a leading method in gene expression prediction, highlighting its potential to significantly enhance the accuracy and reliability of spatial transcriptomics analyses.

### SpaDiT enhances the similarity of predicted gene expression in high-dimensional space

To fully demonstrate the superior ability of the SpaDiT method in gene expression prediction, especially its advantages in maintaining the global and local structural characteristics of gene expression data, we used UMAP technology for visualization analysis for conducting in-depth comparisons with other benchmark methods.

**Figure 3:**
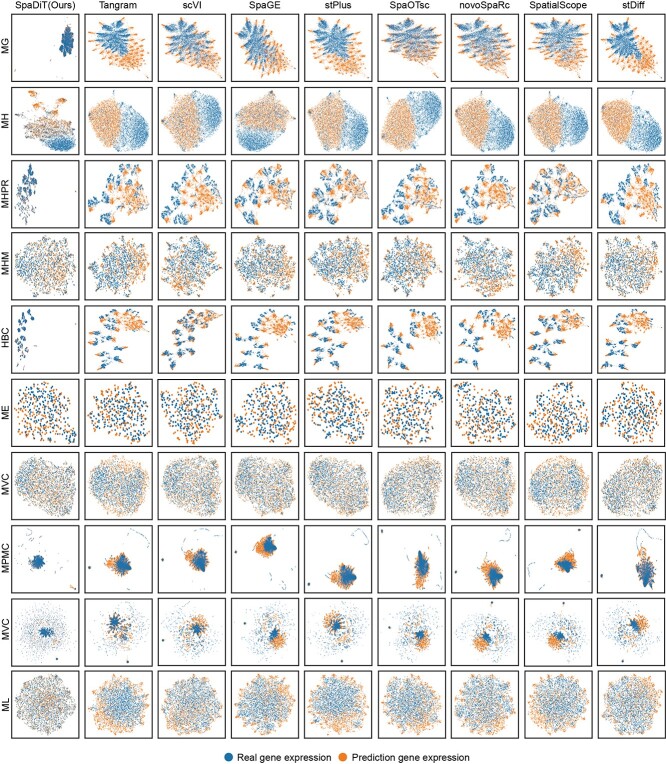
Comparison between SpaDiT and baseline methods: Low-dimensional UMAP visualizations of predicted gene expression vs. real gene expression. In each sub-figure, blue scatter points represent true gene expression, and orange scatter points represent predicted gene expression. The closer the scatter points are, the better the prediction effect of the method. Especially in the SpaDiT method (first column), the blue and orange scatter points almost completely overlap, indicating that its predicted gene expression has a higher similarity with the true gene expression.

As shown in the Fig. 3, we conducted an analysis of ten different datasets. The results clearly show that the prediction results of the SpaDiT method (in orange) closely resemble the real gene expression data (in blue), with minimal perceptible deviation. This is in sharp contrast to the prediction results generated by several other methods, such as Tangram, scVI, SpaGE, stPlus, SpaOTsc, novoSpaRc, SpatialScope, and stDiff. Although the prediction results of these methods have their own focuses, compared with SpaDiT, they all fail to accurately capture the structural characteristics of real gene data and exhibit significant deviations. In addition, the UMAP analysis further underscores SpaDiT’s superiority in maintaining data integrity, enabling it to accurately simulate complex biological information.

### SpaDiT preserves the similarity between genes

To fully demonstrate the accuracy of the SpaDiT method in predicting gene expression, we employed hierarchical clustering to visualize the similarity between the predicted genes and the true gene labels, and compared the results with those from other benchmark methods.

First, we calculated the Euclidean distance between each pair of genes in the gene expression matrix predicted by each method to reflect the similarity of the expression patterns of two genes: the smaller the distance, the higher the similarity. After calculating the distance of all gene pairs, we used hierarchical clustering to sort these genes to ensure that the genes within the cluster show the greatest similarity. With this sorting, we can reorganize the rows and columns of the distance matrix so that similar genes are adjacent to each other in the heat map.

**Figure 4:**
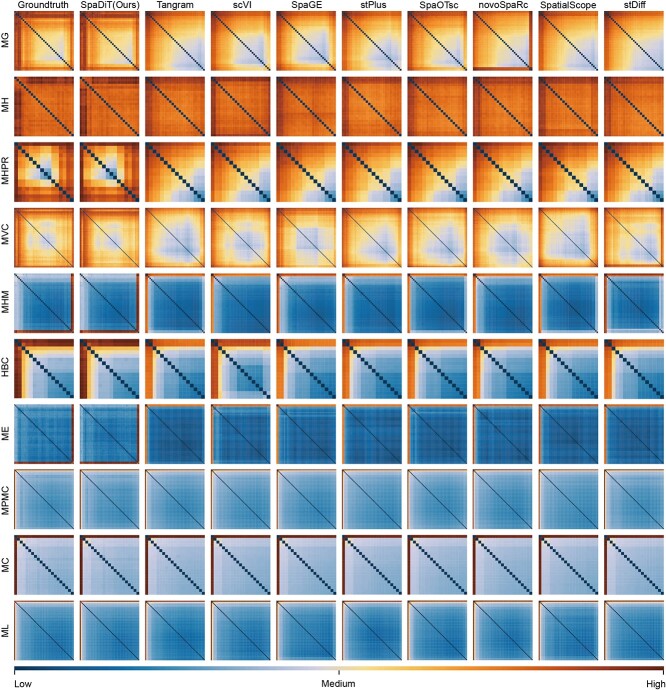
Visualization of the prediction performance of various baseline methods. In order to fully demonstrate the accuracy of the SpaDiT method in gene expression prediction, we used a hierarchical clustering method to visualize the similarity between the predicted genes and the true gene labels in the form of a heat map. The closer the heat map is to the true label, the higher the prediction accuracy of the method. The first column shows the clustering results of the true gene label. As can be seen from the figure, the prediction results of the SpaDiT method are very close to the true label, demonstrating its high accuracy in gene expression prediction.

As shown in the Fig. 4, the first column of the figure visualizes the true gene labels after clustering. The closer the predicted gene heat map is to the true labels, the higher the prediction accuracy of the method. As evident from the figure, the prediction results of the SpaDiT method are very close to the true labels, demonstrating its high accuracy in predicting gene expression.

In addition, we have made hierarchical clustering for spots to discuss whether the prediction methods of different cluster targets are equally effective. The result can be found in the Supplementary Figure S1.

### SpaDiT accurately predicts ST Spatial Patterns

**Figure 5:**
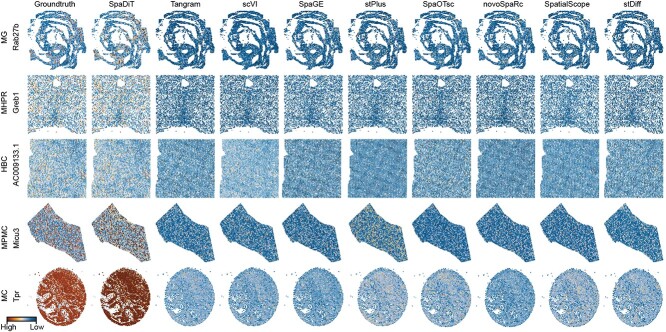
Predicted expression abundance of genes with known spatial patterns in four datasets. Each column corresponds to a gene with a clear spatial pattern. The first column represents the spatial pattern genes with true labels. Subsequent columns show the corresponding predicted expression patterns obtained by using SpaDiT, Tangram, scVI, SpaGE, stPlus, SpaOTsc, novoSpaRc, SpatialScope, and stDiff.

In addition to quantitatively evaluating the gene expression similarity between the true genes of ST and the genes predicted by ST, we also visually demonstrate the consistency of spatial patterns in Fig. 5.

Due to limited space, we selected five datasets with clear spatial patterns: MG, MHPR, HBC, MPMC, and MC to illustrate the consistency of the spatial patterns between the genes predicted by the methods and the true labels. We display the predicted genes with the highest Pearson correlation coefficient (PCC) values in the datasets. The other five datasets not shown can be found in the Supplementary Figure S2.

As illustrated in Fig. 5, in the MG dataset, SpaDiT restores the overall spatial pattern more accurately, followed by stDiff and stPlus, while the other methods show less obvious spatial contours in the upper right part. In the MHPR dataset, SpaDiT provides more accurate predictions in the middle part, while the high expression area and low expression area of other methods appear somewhat chaotic. In the HBC and MPMC datasets, all methods predict relatively accurate spatial patterns, but SpaDiT is the method with expression value predictions closest to the true labels. In the MC dataset, SpaDiT has a clear spatial recognition contour for the lower half, which is closest to the actual situation, while other methods are more blurred at the boundary.

### Robustness evaluation of SpaDiT across various sampling rates

**Figure 6:**
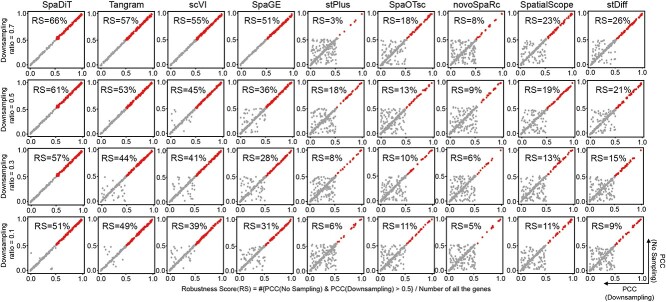
Robustness of prediction accuracy for original data and data with different downsampling rates for the MH dataset. PCC of the spatial distribution of transcripts predicted from the original data and the MH dataset at different downsampling ratios. The PCC values of red transcripts are greater than 0.5 for both the original data and the downsampled data. The proportion of red transcripts in all transcripts is defined as the “robustness score” (RS).

In our study, the sparsity of the ten dataset pairs varies. Most of the datasets are highly sparse spatial transcriptomic data, except for the MH dataset, which has a sparsity of 6.7%. To test the ability of SpaDiT to resist data sparsity, we downsampled the expression matrix of MH’s spatial transcriptomics data to simulate different high-sparse data. To quantify the stability of the SpaDiT and its ability to resist data sparsity, we counted the percentage of genes with a prediction accuracy (PCC) greater than 0.5 in both the original data and the downsampled data, defined as the Robustness Score (RS). As shown in the Fig. 6, the red points represent genes with a PCC value greater than 0.5, and the gray points represent genes with a PCC value less than 0.5. We tested different downsampling rates: 0.1, 0.3, 0.5, and 0.7, and found that the stability scores of all methods decreased with the increase of data sparsity, while the stability score of SpaDiT was always higher than that of other baseline methods. In addition, we compared the changing trends of model performance under different sampling rates and different sparsity levels on ten datasets. For detailed results on other datasets, please refer to the Supplementary Figure S3.

### Downstram analysis of the predicted gene expression on the MHPR dataset

To better demonstrate the significance of the SpaDiT method for biological discovery, we performed downstream analysis on the MHPR dataset, including: cell type annotation, cell clustering, enrichment analysis of predicted genes, and identification of spatially variable genes.

We performed cell type annotation using the scType [39, 40] method, based on the gene expression predicted by SpaDiT and all baselines. The accuracy of the cell type prediction was assessed by calculating the F1-Score between the predicted cell types and the true labels. As shown in Figure S4(A), four types of F1-Scores were computed: (i) the F1-Score for the true gene expression values obtained through sequencing (blue), (ii) the F1-Score combining true sequencing values and predicted expression (green), (iii) the F1-Score for predicted gene expression only (red), and (iv) the F1-Score for random sampling (yellow). A higher F1-Score indicates greater prediction accuracy. SpaDiT achieved the highest accuracy in gene expression prediction across all tested methods.

We employed the Leiden algorithm to cluster cells based on the predicted gene expression. Figure S4(B) and Figure S4(C) presents the results of the clustering analysis, where we used the Adjusted Rand Index (ARI) to evaluate the quality of the clustering against the true cell type labels. SpaDiT displayed superior performance in accurately distinguishing cell types compared to other methods. This accurate clustering is crucial for understanding cell heterogeneity, discovering novel cell types, and gaining deeper insights into tissue composition and function. It also aids in studying the interactions between cells and the functional differentiation of tissues, contributing to our understanding of tissue physiology.

To explore the biological significance of the predicted genes, we conducted gene enrichment analysis using the KEGG and GOBP databases. As depicted in Figure S4(D), several enriched pathways were identified, particularly those related to myelination, nervous system development, and central nervous system development. These pathways are closely linked to the biological functions of the hypothalamic preoptic region, a key area in the mammalian brain that regulates behaviors such as thermoregulation, sleep, and reproduction. The enrichment of neural development and myelination pathways highlights the regulatory roles of the predicted genes in neuronal development and maintenance.

We used the SpatialDE [41] method to identify spatially variable genes (SVG) among the predicted genes. As shown in Figure S4(E), the top six genes we displayed showed different spatial expression patterns in the preoptic area of the hypothalamus, indicating functional heterogeneity in this region. More details could be found in Supplementary Materials.

### Ablation studies

To verify the effectiveness of our proposed method, we conducted detailed ablation experiments on each module of SpaDiT, including the network backbone of SpaDiT, Condition Embedding, and Latent Embedding.

In our proposed, SpaDiT, the main innovation involves using spatial transcriptomics (ST) and single-cell (SC) common genes to concatenate by gene in latent embedding. We use the known part (concatenated SC gene) to infer the gene expression of the unknown part (ST gene to be predicted). This approach enables the model to learn the similarity between different spots and cells across genes. Additionally, the Condition module utilizes the overall SC data as the prior condition to guide the model’s generation process. To verify the effectiveness of the proposed method, we conducted ablation experiments on these two modules separately.

The specific experimental results are shown in Table 3. First, for the Condition module ($\psi $), to verify the effectiveness of the Attention mechanism, we replaced Attention with a simple MLP (Part: w/o Flash-Attention). We found that the performance of the model dropped significantly across ten datasets. Further, to verify the effectiveness of the Condition module ($\psi $) (Part: w/o Condition $\psi $), we replaced the output of the entire part with a vector of all zeros. We observed that compared to replacing Attention, the performance of the model further declined. Additionally, to verify the effectiveness of the overall SC data as a priori conditions (Part: w/ Common Gene in $\psi $), we replaced the overall SC data with SC that only retained the common genes. We found that the performance also declined compared to the overall SC. Finally, to verify the effectiveness of the splicing of the common genes (Part: w/o Concat in $\phi $), we removed this part and found that the performance significantly declined. Therefore, we conclude that the method we proposed is highly effective. In addition, we also tried using Condition modules with different Condition methods.

**Table 3:** Ablation study of Latent Embedding module.

	MG	MH	MHPR	MVC	MHM
**SpaDiT(Ours)**	**0.514$\pm $0.032**	**0.553$\pm $0.057**	**0.506$\pm $0.038**	**0.572$\pm $0.033**	**0.553$\pm $0.037**
w/o Flash-Attention	0.439$\pm $0.092	0.485$\pm $0.027	0.431$\pm $0.028	0.429$\pm $0.013	0.415$\pm $0.017
w/o Condition $\psi $	0.383$\pm $0.094	0.336$\pm $0.115	0.404$\pm $0.161	0.394$\pm $0.066	0.318$\pm $0.013
w/ Common Gene in $\psi $	0.483$\pm $0.126	0.503$\pm $0.008	0.437$\pm $0.125	0.533$\pm $0.161	0.489$\pm $0.088
w/o Concat in $\phi $	0.462$\pm $0.093	0.501$\pm $0.140	0.432$\pm $0.020	0.489$\pm $0.076	0.485$\pm $0.042
	HBC	ME	MPMC	MC	ML
**SpaDiT(Ours)**	**0.613$\pm $0.024**	**0.589$\pm $0.060**	**0.488$\pm $0.033**	**0.564$\pm $0.026**	**0.619$\pm $0.024**
w/o Flash-Attention	0.431$\pm $0.034	0.422$\pm $0.021	0.425$\pm $0.013	0.438$\pm $0.019	0.459$\pm $0.033
w/o Condition module: $\psi $	0.407$\pm $0.053	0.376$\pm $0.169	0.401$\pm $0.050	0.417$\pm $0.108	0.423$\pm $0.022
w/ Common Gene in $\psi $	0.537$\pm $0.032	0.426$\pm $0.142	0.411$\pm $0.083	0.503$\pm $0.050	0.526$\pm $0.062
w/o Concat in $\phi $	0.407$\pm $0.128	0.512$\pm $0.161	0.311$\pm $0.106	0.489$\pm $0.074	0.503$\pm $0.114

For the Condition Embedding part in Table 4, we compared four different Condition embedding methods: MLP, PCA, Attention, and Flash-Attention and used AS as the evaluation metric. As can be seen from the table, the performance of condition embedding using Attention (Attention and Flash-Attention) is the best. The reason may be that the attention mechanism can well capture the importance between genes, so that it can well guide the model to generate more accurate gene expression.

**Table 4:** Ablation study of Different Condition Embedding.

MLP	PCA	Attention	Flash-Attention	MG	MH	MHPR	MVC	MHM
$\checkmark $	$\times $	$\times $	$\times $	0.439$\pm $0.092	0.485$\pm $0.027	0.431$\pm $0.028	0.429$\pm $0.013	0.415$\pm $0.017
$\times $	$\checkmark $	$\times $	$\times $	0.466$\pm $0.027	0.496$\pm $0.066	0.464$\pm $0.023	0.421$\pm $0.027	0.463$\pm $0.022
$\times $	$\times $	$\checkmark $	$\times $	0.513$\pm $0.031	0.507$\pm $0.053	0.518$\pm $0.007	0.538$\pm $0.012	0.507$\pm $0.016
$\times $	$\times $	$\times $	$\checkmark $	**0.514$\pm $0.032**	**0.520$\pm $0.057**	**0.531$\pm $0.038**	**0.506$\pm $0.033**	**0.512$\pm $0.037**
MLP	PCA	Attention	Flash-Attention	HBC	ME	MPMC	MC	ML
$\checkmark $	$\times $	$\times $	$\times $	0.431$\pm $0.034	0.422$\pm $0.021	0.425$\pm $0.013	0.438$\pm $0.019	0.459$\pm $0.033
$\times $	$\checkmark $	$\times $	$\times $	0.496$\pm $0.029	0.476$\pm $0.018	0.466$\pm $0.017	0.451$\pm $0.017	0.476$\pm $0.017
$\times $	$\times $	$\checkmark $	$\times $	0.506$\pm $0.013	0.501$\pm $0.029	0.486$\pm $0.019	0.509$\pm $0.022	0.483$\pm $0.046
$\times $	$\times $	$\times $	$\checkmark $	**0.531$\pm $0.024**	**0.509$\pm $0.060**	**0.508$\pm $0.033**	**0.524$\pm $0.026**	**0.509$\pm $0.024**

For the backbone network part, we used three different network backbones and used AS as the evaluation metric. As shown in the Table 5, we compared three different network architectures: U-Net, Mamba, and Transformer. It is worth noting that the model using Transformer as the network backbone has the best performance, which further proves the importance of Transformer and verifies the superiority of SpaDiT.

**Table 5:** Result of different network backbone.

	MG	MH	MHPR	MVC	MHM
Backbone w/Unet	0.454$\pm $0.011	0.453$\pm $0.011	0.477$\pm $0.013	0.482$\pm $0.011	0.466$\pm $0.011
Backbone w/Mamba	0.477$\pm $0.008	0.471$\pm $0.026	0.475$\pm $0.014	0.474$\pm $0.011	0.461$\pm $0.102
Backbone w/Transformer	**0.514$\pm $0.032**	**0.553$\pm $0.057**	**0.506$\pm $0.038**	**0.572$\pm $0.033**	**0.553$\pm $0.037**
	HBC	ME	MPMC	MC	ML
Backbone w/Unet	0.478$\pm $0.012	0.470$\pm $0.010	0.458$\pm $0.013	0.470$\pm $0.010	0.487$\pm $0.013
Backbone w/Mamba	0.462$\pm $0.086	0.489$\pm $0.051	0.421$\pm $0.022	0.478$\pm $0.015	0.488$\pm $0.021
Backbone w/Transformer	**0.613$\pm $0.024**	**0.589$\pm $0.060**	**0.488$\pm $0.033**	**0.564$\pm $0.026**	**0.619$\pm $0.024**

## Discussion

In this paper, we present SpaDiT, a novel approach to predict unmeasured genes in spatial transcriptomics (ST) data. Methodologically, SpaDiT is significantly different from existing ensemble techniques. While traditional approaches primarily enhance ST data by aligning ST data to similar cells within a reference scRNA-seq dataset, SpaDiT employs a diffusion-based generative model that utilizes the inherent relationships within the gene expression data. This approach enables it to precisely model and generate spatial gene expression patterns.

SpaDiT, as a conditional diffusion model, employs noise addition and denoising stages to learn complex relationships from scRNA-seq data. In the inference stage, SpaDiT incorporates raw ST data during the denoising process, resulting in accurate predictions of spatial gene expression. The application of diffusion models in genomics, especially transcriptomics, is relatively new, marking this as a largely unexplored area. We assessed SpaDiT using ten ST datasets, employing multiple metrics to evaluate performance, gene spatial structure, and gene similarity. The results show that SpaDiT not only maintains the intricate topology inherent in cell layout but also excels in accurately aligning predicted gene expression with actual data, demonstrating its robustness and accuracy in reproducing spatial patterns. These features highlight the utility of SpaDiT in enhancing the resolution and richness of ST data analysis. We also performed ablation experiments on various modules of SpaDiT (network backbone and conditional embedding) to demonstrate the effectiveness of our approach.

Future research may combine SpaDiT’s diffusion-based approach with traditional similarity-based methods to enhance the accuracy of ST data predictions. These advances may significantly improve the analysis and interpretation of ST data, potentially setting new standards in the field. It is important to acknowledge the potential limitations. For example, when ST data lack sufficient markers to accurately identify cell types, SpaDiT’s efficacy may be diminished, similar to other methods. This is due to the reliance on existing gene expression signals to guide the prediction process, potentially resulting in inaccuracies if the initial data are too sparse or ambiguous.

In terms of scalability and computational efficiency, SpaDiT is designed to handle large-scale spatial transcriptomics data and multimodal datasets through batch processing techniques, which effectively manage memory usage while maintaining high processing speeds. This approach enables SpaDiT to process large datasets without performance degradation, optimizing memory usage to accommodate larger datasets even with limited hardware resources. Our experiments demonstrate that SpaDiT’s runtime scales linearly with increasing dataset sizes. For a medium-sized dataset (about 5000 spots and 3000 genes), SpaDiT requires approximately 20GB of GPU memory and takes around 9 GPU hours for training and inference. This linear scalability pattern persists with larger datasets, underscoring SpaDiT’s ability to efficiently handle increasing data volumes while benefiting from improved hardware configurations to further reduce processing time.

This underscores the need for improvements in handling datasets with limited information, ensuring that SpaDiT can adapt to various levels of data completeness and quality.

**Key Points**

- In this study, we propose SpaDiT, a deep learning method that utilizes a conditional diffusion generative model to synthesize scRNA-seq data and ST data to predict undetected genes.
- We utilize scRNA-seq as a prior condition and integrate it into the diffusion model through the attention mechanism to guide the model in learning the relationship between ST and scRNA-seq. At the same time, the common genes in ST and scRNA-seq are concatenated as the “token” input of the model, so that SpaDiT can learn multi-scale feature information and more accurately predict unknown genes.
- Our method was compared with competing methods on ten real ST and scRNA-seq datasets. The results show that, compared with the most advanced methods, our method demonstrates significant improvements in all five evaluation metrics in predicting gene expression. In addition, the genes predicted by our proposed SpaDiT effectively maintain high-dimensional similarity with the real labels, clearly restoring the spatial patterns between genes and the similarities between genes.

## Supplementary Material

Supplementary_Materials_Final_bbae571
