# Supplementary material for: SpaDiT: diffusion transformer for spatial gene expression prediction using scRNA-seq
Source: Brief Bioinform. 2024 Nov 7;25(6):bbae571. doi: 10.1093/bib/bbae571 (PMC11541600; doi:10.1093/bib/bbae571)
Supplement: Supplementary_Materials_Final_bbae571 [file supplementary_materials_final_bbae571.pdf]

Supplementary Materials for

SpaDiT: Diffusion Transformer for Spatial Gene Expression Prediction using scRNA-seq

Supplementary Notes

1. Overview of DDPM and score-based diffusion model

In the realm of denoising diffusion probabilistic models [1, 2], consider the task of learning a model distribution $p_\theta(\mathbf{x}_0)$ that closely approximates a given data distribution $q(\mathbf{x}_0)$. Suppose we have a sequence of latent variables $\mathbf{x}_t$ for $t = 1, \dots, T$, existing within the same sample space as $\mathbf{x}_0$, which is denoted as $\mathcal{X}$. DDPMs are latent variable models that are composed of two primary processes: the forward process and the reverse process. The forward process is defined by a Markov chain, described as follows:

$$\begin{aligned} q(\mathbf{x}_{1:T}|\mathbf{x}_0) &:= \prod_{t=1}^{T} q(\mathbf{x}_t|\mathbf{x}_{t-1}), \\ \text{where } q(\mathbf{x}_t|\mathbf{x}_{t-1}) &:= \mathcal{N}(\sqrt{1-\beta_t}\mathbf{x}_{t-1}, \beta_t \mathbf{I}), \end{aligned} \tag{1}$$

and the variable β_t is a small positive constant indicative of a noise level. The sampling of x_t can be described by the closed-form expression $q(x_t|x_0) = \mathcal{N}(x_t; \sqrt{\alpha_t}x_0, (1-\alpha_t)\mathbf{I})$, where $\hat{\alpha}_t := 1 - \beta_t$ and α_t is the cumulative product $\alpha_t := \prod_{i=1}^{t} \hat{\alpha}_i$. Consequently, x_t is given by the equation $x_t = \sqrt{\alpha_t}x_0 + (1-\alpha_t)\epsilon$, with $\epsilon \sim \mathcal{N}(0, \mathbf{I})$. In contrast, the reverse process aims to denoise x_t to retrieve x_0, a process which is characterized by the ensuing Markov chain:

$$\begin{aligned} p_\theta(\mathbf{x}_{0:T}) &:= p(\mathbf{x}_T) \prod_{t=1}^{T} p_\theta(\mathbf{x}_{t-1}|\mathbf{x}_t), \quad \mathbf{x}_T \sim \mathcal{N}(0, \mathbf{I}), \\ p_\theta(\mathbf{x}_{t-1}|\mathbf{x}_t) &:= \mathcal{N}(\mathbf{x}_{t-1}; \mu_\theta(\mathbf{x}_t, t), \sigma_\theta^2(\mathbf{x}_t, t)\mathbf{I}), \end{aligned} \tag{2}$$

$$\begin{aligned} &\mu_\theta(\mathbf{x}_t, t) = \frac{1}{\alpha_t}\left(\mathbf{x}_t - \frac{\beta_t}{\sqrt{1-\alpha_t}}\epsilon_\theta(\mathbf{x}_t, t)\right), \\ &\sigma_\theta(\mathbf{x}_t, t) = \beta_t^{1/2}, \\ &\text{where } \beta_t = \begin{cases} \frac{1-\bar{\alpha}_{t-1}}{1-\bar{\alpha}_t}\beta_1, & \text{for } t > 1, \\ \beta_1, & \text{for } t = 1, \end{cases} \\ &\text{and } \epsilon_\theta(\mathbf{x}_t, t) \text{ is a trainable denoising function} \end{aligned} \tag{3}$$

But in the score-based model, it is slightly different from DDPM. Its forward process is based on a forward stochastic differential equation (SDE), $x(t)$ with $t \in [0, T]$, defined as:

$$\mathrm{d}x(t) = f(x(t), t)\mathrm{d}t + g(t)\mathrm{d}w, \tag{4}$$

where w is the standard Wiener process (Brownian motion), $f(\cdot, t) : \mathbb{R}^d \to \mathbb{R}^d$ is a vector-valued function called the drift coefficient of $x(t)$. The reverse process is performed via the reverse SDE, first sampling data x_t from $p(x_t)$ and the generate x_0 through the reverse of Eq. (4) as:

$$\mathrm{d}x(t) = [f(x(t), t) - g(t)^2\nabla_x \mathrm{log}p(x_t)]\mathrm{d}t + g(t)\mathrm{d}\bar{w}, \tag{5}$$

where $\bar{w}$ is the standard Wiener process when time flows backwards from T to 0, and $\mathrm{d}t$ is an infinitesimal negative timestep.

The biggest difference between DDPM and score-based model is the estimation of noise equation. In the DDPM and score-based models, the estimation of the noise function is defined as $\epsilon_\theta\,(x_t, t)$ and $s_\theta\,(x_t, t)$:

$$\begin{aligned} s_\theta\,(x_t, t) &= \nabla_x \log p_t(x) = -\frac{x_t - \sqrt{\alpha_t}x_0}{1-\alpha_t}, \\ \epsilon_\theta\,(x_t, t) &= \frac{x_t - \sqrt{\alpha_t}x_0}{\sqrt{1-\alpha_t}}, \\ s_\theta\,(x_t, t) &= -\frac{1}{\sqrt{1-\alpha_t}}\epsilon_\theta\,(x_t, t), \end{aligned} \tag{6}$$

So we get how to convert DDPM to Score-based model in the sampling stage: $s_\theta\,(x_t, t) = -\frac{1}{\sqrt{1-\bar{\alpha}_t}}\epsilon_\theta\,(x_t, t)$.

In the DDPM [2], considering the following specific parameterization of $p_\theta(\mathbf{x}_{t-1}|\mathbf{x}_t)$ are learned from the data by optimizing a variational lower bound:

$$\begin{aligned}\log q(x_0) \geq \mathbb{E}_{q(x_0)}[&\underbrace{\log p_\theta(x_0|x_1)}_{\mathcal{L}_0} - \underbrace{KL\left(q(x_T|x_0)||q(x_T)\right)}_{\mathcal{L}_T} \\ &- \sum_{t=2}^{T}\underbrace{KL\left(q(x_{t-1}|x_t, x_0)||p_\theta(x_{t-1}|x_t)\right)}_{\mathcal{L}_t}],\end{aligned} \tag{7}$$

where $\mathcal{L}_0$ is that given slightly noisy data x_1, correctly reconstruct the original data probability of x_0, $\mathcal{L}_T$ measures the discrepancy between the model distribution $q(x_T|x_0)$ and the prior noise distribution $q(x_T)$ at the last noise level T, $\mathcal{L}_t$ measures how the model handles the denoising process from x_t to x_{t-1} at each time step, encouraging the model to learn how to remove noise at each step. In practice, Eq. (7) can be simplified to the following form:

$$\min_\theta \mathcal{L}(\theta) := \mathbb{E}_{q(\mathbf{x}_0),\epsilon\sim\mathcal{N}(0,\mathbf{I})}\|\epsilon - \epsilon_\theta(\mathbf{x}_t, t)\|_2^2, \tag{8}$$

where $\mathbf{x}_t = \sqrt{\alpha_t}\mathbf{x}_0 + (1-\alpha_t)\epsilon$. The denoising function ϵ_θ is designed to estimate the noise vectior ϵ originally added to the noisy input x_t. Additionally, this training objective also be interpreted as a weighted combination of denoising and score matching, which are techniques commonly employed in the training of score-based generative models.

2. Forward diffusion process

Given a data point sampled from a real data distribution $x_o \sim q(x)$, let us define a *forward diffusion process* in which we add small amount of Gaussian noise to the sample in T steps, producing a sequence of noisy samples $x_1, \ldots, x_T$. The step sizes are controlled by a variance schedule $\{\beta_t \in (0,1)\}_{t=1}^T$.

$$\begin{aligned} q(x_t|x_{t-1}) &= \mathcal{N}(x_t; \sqrt{1-\beta_{t-1}}x_{t-1}, \beta_{t-1}\mathbf{I}) \\ q(x_{1:T}|x_0) &= \prod_{t=1}^{T} q(x_t|x_{t-1}) \end{aligned} \tag{9}$$

The data sample x_0 gradually loss its distinguishable features as the step t becomes larger. Eventually when $T \to \infty$, x_T is equivalent to an isotropic Gaussian distribution. A nice property of the above process is that we can sample x_t at any arbitrary time step t in a closed form using reparameterization trick. Let $\alpha_t = 1 - \beta_t$ and $\bar{\alpha}_t = \prod_{i=1}^t \alpha_i$:

$$\begin{aligned} x_t &= \sqrt{\alpha_t}x_{t-1} + \sqrt{1-\alpha_t}\epsilon_{t-1} \\ &= \sqrt{\alpha_t\alpha_{t-1}}x_{t-2} + \sqrt{1-\alpha_t\alpha_{t-1}}\bar{\epsilon}_{t-2} \\ &\vdots \\ &= \sqrt{\overline{\alpha}_t}x_0 + \sqrt{1-\overline{\alpha}_t}\epsilon \\ q(x_t|x_0) &= \mathcal{N}(x_t; \sqrt{\overline{\alpha}_t}x_0, (1-\overline{\alpha}_t)I) \end{aligned} \tag{10}$$

where$\epsilon_{t-1}, \epsilon_{t-2}, \ldots \sim \mathcal{N}(0, I)$; where$\bar{\epsilon}_{t-2}$merges two Gaussians(*). (*) Recall that when we merge two Gaussians with different variance, $\mathcal{N}(0, \sigma_1^2\mathrm{I})$ and $\mathcal{N}(0, \sigma_2^2\mathrm{I})$, the new distribution is $\mathcal{N}(0, (\sigma_1^2 + \sigma_2^2)\mathrm{I})$. Here the merged standard deviation is $\sqrt{(1-\alpha_t) + \alpha_t(1-\alpha_{t-1})} = \sqrt{1-\alpha_t\alpha_{t-1}}$. Usually, we can afford a larger update step when the sample gets noisier, so $\beta_1 < \beta_2 < \ldots < \beta_T$ and therefore $\bar{\alpha}_1 > \ldots > \bar{\alpha}_T$

3. Connection with stochastic gradient Langevin dynamics in score-base model

Langevin dynamics is a concept from physics, developed for statistically modeling molecular systems. Combined with stochastic gradient descent, stochastic gradient Langevin dynamics can produce samples from a probability density $p(x)$ using only the gradients $\nabla_x \mathrm{log}p(x)$ in a Markov chain of updates:

$$x_t = x_{t-1} + \frac{\delta}{2}\nabla_x \log p(x_{t-1}) + \sqrt{\delta}\epsilon_t, \quad \text{where } \epsilon_t \sim \mathcal{N}(0, I) \tag{11}$$

where δ is the stpe size. When $T \to \infty, \epsilon \to 0, x_T$ equals to the true probability density $p(x)$. Compared to standard SGD, stochastic gradient Langevin dynamics injects Gaussian noise into the parameter updates to avoid collapses into local minima.

4. Reverse diffusion process

If we can reverse the above process and sample from $q(x_{t-1}|x_t)$, we we will be able to recreate the true sample from a Gaussian noise input, $x_T \sim \mathcal{N}(0, \mathrm{I})$. Note that if β_t is small enough, $q(x_{t-1}|x_t)$ will also be Gaussian. Unfortunately, we cannot easily

estimate $q(x_{t-1}|x_t)$ because it needs to use the entire dataset and therefore we need to learn a model p_θ to approximate these conditional probabilities in order to run the reverse diffusion process.

$$\begin{aligned} p_\theta(x_0:T) &= p_\theta(x_T)\prod_{t=1}^{T} p_\theta(x_{t-1}|x_t) \\ p_\theta(x_{t-1}|x_t) &= \mathcal{N}(x_{t-1};\mu_\theta(x_t,t),\Sigma_\theta(x_t,t)) \end{aligned} \tag{12}$$

It is noteworthy that the reverse conditional probability is tractable when conditioned on x_0:

$$q(x_{t-1}|x_t,x_0) = \mathcal{N}(x_{t-1};\mu(x_t,x_0),\beta I) \tag{13}$$

Using Bayes' rule, we have:

$$\begin{aligned} q(x_{t-1}|x_t,x_0) &= \frac{q(x_{t-1},x_t)}{q(x_t|x_0)} \\ &\propto \exp\Bigg(-\frac{1}{2}\Bigg(\frac{(x_t-\sqrt{\alpha_t}x_{t-1})^2}{\beta_t} \\ &+ \frac{(x_{t-1}-\sqrt{\overline{\alpha}_{t-1}}x_0)^2}{1-\overline{\alpha}_{t-1}} - \frac{(x_t-\sqrt{\overline{\alpha}_t}x_0)^2}{1-\overline{\alpha}_t}\Bigg)\Bigg) \\ &= \exp\Bigg(-\frac{1}{2}\Bigg(\frac{x_t^2}{\beta_t} - 2\frac{\sqrt{\alpha_t}x_{t-1}x_t}{\beta_t} \\ &+ \frac{\alpha_t x_{t-1}^2}{\beta_t} - 2\frac{\sqrt{\overline{\alpha}_{t-1}}x_{t-1}x_0}{1-\overline{\alpha}_{t-1}} + \frac{x_{t-1}^2}{1-\overline{\alpha}_{t-1}} + C(x_t,x_0)\Bigg)\Bigg) \\ &= \exp\Bigg(-\frac{1}{2}\Bigg(\left(\frac{\alpha_t}{\beta_t}+\frac{1}{1-\overline{\alpha}_{t-1}}\right)x_{t-1}^2 \\ &- 2\left(\frac{\sqrt{\alpha_t}}{\beta_t}+\frac{\sqrt{\overline{\alpha}_{t-1}}}{1-\overline{\alpha}_{t-1}}\right)x_{t-1}x_t \\ &+ C(x_t,x_0)\Bigg)\Bigg) \end{aligned} \tag{14}$$

where $C(x_t,x_0)$ is some function not involving x_{t-1} and details are omitted. Following the standard Gaussian density function, the mean and variance can be parameterized as follows (recall that $\alpha_t = 1-\beta_t$ and $\bar{\alpha}_t = \prod_{i=1}^{t}\alpha_i$):

$$\begin{aligned} \hat{\beta}_t &= \frac{1}{\left(\frac{\alpha_t}{\beta_t}+\frac{1}{1-\overline{\alpha}_{t-1}}\right)} = \frac{1}{\left(\frac{\alpha_t-\overline{\alpha}_t+\beta_t}{\beta_t(1-\overline{\alpha}_{t-1})}\right)} \\ &= \frac{1-\overline{\alpha}_{t-1}}{\alpha_t-\overline{\alpha}_t+\beta_t} = \frac{1-\overline{\alpha}_{t-1}}{1-\overline{\alpha}_t}\cdot\beta_t, \\ \hat{\mu}(x_t,x_0) &= \left(\frac{\sqrt{\overline{\alpha}_t}x_t}{\beta_t}+\frac{\sqrt{1-\overline{\alpha}_{t-1}}x_0}{1-\overline{\alpha}_{t-1}}\right)\Big/\left(\frac{\alpha_t}{\beta_t}+\frac{1}{1-\overline{\alpha}_{t-1}}\right) \\ &= \left(\frac{\sqrt{\overline{\alpha}_t}x_t}{\beta_t}+\frac{\sqrt{1-\overline{\alpha}_{t-1}}x_0}{1-\overline{\alpha}_{t-1}}\right)\cdot\frac{1-\overline{\alpha}_{t-1}}{1-\overline{\alpha}_t}\cdot\beta_t \\ &= \frac{\sqrt{\overline{\alpha}_t(1-\overline{\alpha}_{t-1})}x_t+\sqrt{\overline{\alpha}_{t-1}\beta_t}x_0}{1-\overline{\alpha}_t}. \end{aligned} \tag{15}$$

We can represent $x_0 = \frac{1}{\sqrt{\overline{\alpha}_t}}\left(x_t-\sqrt{1-\overline{\alpha}_t}\epsilon_t\right)$ and plug into the above equation and obtain:

$$\begin{aligned} \hat{\mu}_t &= \frac{\sqrt{\overline{\alpha}_t(1-\overline{\alpha}_{t-1})}}{1-\overline{\alpha}_t}x_t + \frac{\sqrt{\overline{\alpha}_{t-1}\beta_t}}{1-\overline{\alpha}_t}\frac{1}{\sqrt{\overline{\alpha}_t}}\left(x_t-\sqrt{1-\overline{\alpha}_t}\epsilon_t\right) \\ &= \frac{1}{\sqrt{\alpha_t}}\left(x_t-\frac{1-\alpha_t}{\sqrt{1-\overline{\alpha}_t}}\epsilon_t\right) \end{aligned} \tag{16}$$

such a setup is very similar to VAE and thus we can use the variational lower bound to optimize the negative log-likelihood.

$$\begin{aligned} -\log p_\theta(x_0) &\leq -\log p_\theta(x_0) + D_{KL}(q(x_{1:T}|x_0)\|p_\theta(x_{1:T}|x_0)) \\ &= -\log p_\theta(x_0) + \mathbb{E}_{x_{1:T}\sim q(x_{1:T}|x_0)}\left[\log \frac{q(x_{1:T}|x_0)}{p_\theta(x_{0:T})\ /p_\theta(x_0)}\right] \\ &= -\log p_\theta(x_0) + \mathbb{E}_q\left[\log \frac{q(x_{1:T}|x_0)}{p_\theta(x_{0:T})} + \log p_\theta(x_0)\right] \\ &= \mathbb{E}_q\left[\log \frac{q(x_{1:T}|x_0)}{p_\theta(x_{0:T})}\right] \\ \text{Let } L_{VLB} &= \mathbb{E}_{q(x_{0:T})}\left[\log \frac{q(x_{1:T}|x_0)}{p_\theta(x_{0:T})}\right] \geq -\mathbb{E}_{q(x_0)} \log p_\theta(x_0) \end{aligned} \tag{17}$$

It is also straightforward to get the same result using Jensen's inequality. Say we want to minimize the cross entropy as the learning objective,

$$\begin{aligned} L_{CE} &= -\mathbb{E}_{q(x_0)} \log p_\theta(x_0) \\ &= -\mathbb{E}_{q(x_0)} \log \left(\int p_\theta(x_0:T)\, dx_1:T\right) \\ &= -\mathbb{E}_{q(x_0)} \log \left(\int \frac{q(x_1:T|x_0)p_\theta(x_0:T)}{q(x_1:T|x_0)}\, dx_1:T\right) \\ &= -\mathbb{E}_{q(x_0)} \log \left(\mathbb{E}_{q(x_1:T|x_0)}\left[\frac{p_\theta(x_0:T)}{q(x_1:T|x_0)}\right]\right) \\ &\leq -\mathbb{E}_{q(x_0:T)} \log \frac{p_\theta(x_0:T)}{q(x_1:T|x_0)} \\ &= \mathbb{E}_{q(x_0:T)} \log \frac{q(x_1:T|x_0)}{p_\theta(x_0:T)} \\ &= L_{VLB} \end{aligned} \tag{18}$$

To convert each term in the equation to be analytically computable, the objective can be further rewritten to be a combination of several KL-divergence and entropy terms:

$$\begin{aligned} &L_{VLB} = \mathbb{E}_{q(x_0:T)}\left[\log \frac{q(x_1:T|x_0)}{p_\theta(x_0:T)}\right] \\ &= \mathbb{E}_q\left[\log \frac{q(x_T|x_0)}{p_\theta(x_T)} \prod_{t=1}^{T} \frac{q(x_{t-1}|x_t)}{p_\theta(x_{t-1}|x_t)}\right] \\ &= \mathbb{E}_q\left[-\log p_\theta(x_T) + \sum_{t=1}^{T} \log \frac{q(x_{t-1}|x_t)}{p_\theta(x_{t-1}|x_t)}\right] \\ &= \mathbb{E}_q\left[-\log p_\theta(x_T) + \log \frac{q(x_{T-1}|x_T)}{p_\theta(x_{T-1}|x_T)} + \log \frac{q(x_1|x_2)}{p_\theta(x_1|x_2)}\right] \\ &= \mathbb{E}_q\left[-\log p_\theta(x_T) + \sum_{t=2}^{T} \log \frac{q(x_{t-1}|x_t, x_0)}{p_\theta(x_{t-1}|x_t)} + \log \frac{q(x_1|x_0)}{p_\theta(x_1|x_0)}\right] \\ &= \mathbb{E}_q\left[-\log p_\theta(x_T) + \sum_{t=2}^{T} \log \frac{q(x_{t-1}|x_t, x_0)}{p_\theta(x_{t-1}|x_t)}\right] + \mathbb{E}_q\left[\log \frac{q(x_1|x_0)}{p_\theta(x_1|x_0)}\right] \\ &= \mathbb{E}_q\left[-\log p_\theta(x_T) + \sum_{t=2}^{T} \log \frac{q(x_{t-1}|x_t, x_0)}{p_\theta(x_{t-1}|x_t)}\right] - \mathbb{E}_{q(x_0)} \log p_\theta(x_0|x_1) \\ &= \mathbb{E}_q \underbrace{\left[\log \frac{q(x_T|x_0)}{p_\theta(x_T)}\right]}_{L_T} + \sum_{t=2}^{T} \mathbb{E}_q \underbrace{\left[D_{KL}(q(x_{t-1}|x_t, x_0)||p_\theta(x_{t-1}|x_t))\right]}_{L_{t-1}} - \\ &\mathbb{E}_{q(x_0)} \underbrace{\log p_\theta(x_0|x_1)}_{L_0} \end{aligned} \tag{19}$$

Let's label each component in the variational lower bound loss separately:

$$\begin{aligned} L_{VLB} &= L_T + L_{T-1} + \cdots + L_0 \\ \text{where} \quad L_T &= D_{KL}(q(x_T|x_0)\|p_\theta(x_T)), \\ L_t &= D_{KL}(q(x_t|x_{t+1}, x_0)\|p_\theta(x_t|x_{t+1})) \\ &\text{for } 1 < t < T, \\ L_0 &= -\log p_\theta(x_0|x_1). \end{aligned} \tag{20}$$

Every KL term in L_{VLB} (except for L_0) compares two Gaussian distributions and therefore they can be computed in closed form. L_T is constant and can be ignored during training because q has no learnable parameters and $x_T$is a Gaussian noise. The DDPM models L_0 using a separate discrete decoder derived from $\mathcal{N}(\mathbf{x_0}; \mu_\theta(\mathbf{x_1}, 1), \Sigma_\theta(\mathbf{x_1}, 1))$.

5. Parameterization of L_t for Training Loss

Recall that we need to learn a neural network to approximate the conditioned probability distributions in the reverse diffusion process, $p_\theta(x_{t-1}|x_t) = \mathcal{N}(\mathbf{x_{t-1}}; \mu_\theta(\mathbf{x_t}, t), \Sigma_\theta(\mathbf{x_t}, t))$. We would like to train μ_θ to predict $\hat{\mu}_t = \frac{1}{\sqrt{\alpha_t}}\left(x_t - \frac{1-\alpha_t}{\sqrt{1-\bar{\alpha}_t}}\epsilon_t\right)$. Because x_t is available as input at training time, we can reparameterize the Gaussian noise term instead to make it predict ϵ_t from the input x_t at time step t:

$$\begin{aligned} \mu_\theta(x_t, t) &= \frac{1}{\sqrt{\alpha_t}}\left(x_t - \frac{1-\alpha_t}{\sqrt{1-\alpha_t}}\epsilon_\theta(x_t, t)\right) \\ \text{Thus } x_{t-1} &\sim \mathcal{N}\left(x_{t-1}; \frac{1}{\sqrt{\alpha_t}}\left(x_t - \frac{1-\alpha_t}{\sqrt{1-\alpha_t}}\epsilon_\theta(x_t, t)\right), \Sigma_\theta(x_t, t)\right) \end{aligned} \tag{21}$$

The loss term L_t is parameterized to minimize the difference from $\hat{\mu}$:

$$\begin{aligned} L_t &= \mathbb{E}_{x_0, \epsilon_t}\left[\frac{1}{2}\frac{\|\mu_\theta(x_t, x_0) - \mu_\theta(x_t, t)\|^2}{\|\Sigma_\theta(x_t, t)\|_2^2}\right] \\ &= \mathbb{E}_{x_0, \epsilon_t}\left[\frac{1}{2}\frac{1}{\|\Sigma_\theta(x_t, t)\|_2^2}\left\|\frac{1}{\sqrt{\alpha_t}}(x_t - \frac{1-\alpha_t}{\sqrt{1-\alpha_t}}\epsilon_t)\right.\right. \\ &\qquad \left.\left. - \frac{1}{\sqrt{\alpha_t}}(x_t - \frac{1-\alpha_t}{\sqrt{1-\alpha_t}}\epsilon_\theta(x_t, t))\right\|^2\right] \\ &= \mathbb{E}_{x_0, \epsilon_t}\left[\frac{1}{2\alpha_t(1-\alpha_t)}\frac{1}{\|\Sigma_\theta(x_t, t)\|_2^2}\|\epsilon_t - \epsilon_\theta(x_t, t)\|^2\right] \\ &= \mathbb{E}_{x_0, \epsilon_t}\left[\frac{1}{2\alpha_t(1-\alpha_t)}\frac{1}{\|\Sigma_\theta(x_t, t)\|_2^2}\right. \\ &\qquad \left. \times \|\epsilon_t - \epsilon_\theta(\sqrt{\alpha_t}x_0 + \sqrt{1-\alpha_t}\epsilon_t, t)\|^2\right] \end{aligned} \tag{22}$$

6. Connection with noise-conditioned score networks (NCSN) in score-based model

Song & Ermon (2019) proposed a score-based generative modeling method where samples are produced via *Langevin dynamics* using gradients of the data distribution estimated with score matching. The score of each sample $\mathbf{x}$'s density probability is defined as its gradient $\nabla_{\mathbf{x}} \log q(\mathbf{x})$. A score network $s_\theta : \mathbb{R}^D \to \mathbb{R}^D$ is trained to estimate it, $s_\theta(\mathbf{x}) \approx \nabla_{\mathbf{x}} \log q(\mathbf{x})$.

To make it scalable with high-dimensional data in the deep learning setting, they proposed to use either *denoising score matching* (Vincent, 2011) or *sliced score matching* (use random projections; Song et al., 2019). Denoising score matching adds a pre-specified small noise to the data $\tilde{q}(\mathbf{x})$ and estimates $q(\mathbf{x})$ with score matching.

Recall that Langevin dynamics can sample data points from a probability density distribution using only the score $\nabla_{\mathbf{x}} \log q(\mathbf{x})$ in an iterative process.

However, according to the manifold hypothesis, most of the data is expected to concentrate in a low dimensional manifold, even though the observed data might look only arbitrarily high-dimensional. It brings a negative effect on score estimation since the data points cannot cover the whole space. In regions where data density is low, the score estimation is less reliable. After adding a small Gaussian noise to make the perturbed data distribution cover the full space $\mathbb{R}^D$, the training of the score estimator network becomes more stable. Song & Ermon (2019) improved it by perturbing the data with the noise of *different levels* and train a noise-conditioned score network to *jointly* estimate the scores of all the perturbed data at different noise levels.

The schedule of increasing noise levels resembles the forward diffusion process. If we use the diffusion process annotation, the score approximates $s_\theta(x_t, t) \approx \nabla_{\mathbf{x}} \log q(\mathbf{x})$. Given a Gaussian distribution $\mathcal{N}(\mu, \sigma^2)$, we can write the derivative of the logarithm of its density function as $\nabla_{\mathbf{x}} \log p(\mathbf{x}) = \nabla_{\mathbf{x}}\left(-\frac{(\mathbf{x}-\mu)^2}{2\sigma^2}\right) = -\frac{\mathbf{x}-\mu}{\sigma^2} = -\frac{\mathbf{x}-\mu}{\sigma^2} - \epsilon$ where $\epsilon \sim \mathcal{N}(0, I)$.Recall that$q(\mathbf{x}_t) \sim$

$\mathcal{N}\left(\sqrt{\alpha_t}\mathbf{x}_0, (1-\alpha_t)I\right)$ and therefore,

$$\begin{aligned} s_\theta(\mathbf{x}_t, t) &\approx \nabla_{\mathbf{x}} \log q(\mathbf{x}) = \mathbb{E}_{q(\mathbf{x}_0)}\left[\nabla_{\mathbf{x}} \log q(\mathbf{x}_t|\mathbf{x}_0)\right] \\ &= \mathbb{E}_{q(\mathbf{x}_0)}\left[-\frac{\epsilon_t(\mathbf{x}_t, t)}{\sqrt{1-\alpha_t}}\right] = -\frac{\epsilon_t(\mathbf{x}_t, t)}{\sqrt{1-\alpha_t}}. \end{aligned} \tag{23}$$

7. Implementation Details of SpaDiT

In this section, we offer a comprehensive description of the experimental configurations utilized in our study. We assess the performance of baseline methods by employing the default hyperparameters and model dimensions that were originally proposed. Details on these hyperparameters are thoroughly documented in Section S1 of this paper.

We employ an exponential moving average (EMA) of the model parameters with a decay rate of 0.926. The model is trained on a single NVIDIA RTX 4090, 24 GB, using the AdamW optimizer in PyTorch. For evaluating baseline methods, we adhere to their original hyperparameters and model sizes.

Furthermore, based on insights from recent studies such as [3] and [4], it has been observed that a gradual reduction in the noise levels, specifically through a gentle decay of the parameter α_t, can enhance the quality of the generated samples. To incorporate this finding into our experimentation, we have implemented a quadratic decay schedule for α_t across different noise levels. This approach allows us to systematically evaluate the impact of modifying the noise decay trajectory on the performance and output quality of our model.

$$\beta_t = \left(\frac{T-t}{T-1}\sqrt{\beta_1} + \frac{t-1}{T-1}\sqrt{\beta_T}\right)^2 \tag{24}$$

Table S1. Hyperparameter details of SpaDiT.

Datasets	Batchsize	Depth	Diffusion Step	Epoch	Backbone Head	Hidden size	Learning Rate	Mask_zero_ratio	Mask_non_zero_ratio	Latent layers
MG [5]	512	32	1000	3000	32	1024	3e-4	0.4	0.3	3
MH [6]	512	32	1000	4000	16	2048	2e-6	0.3	0.1	2
MHPR [7]	1024	16	2000	3000	8	1024	1e-4	0.1	0.2	3
MVC [8]	1024	64	2000	4000	64	1024	3e-6	0.3	0.2	4
MHM [9]	1024	64	2000	4000	64	1024	3e-6	0.3	0.2	4
HBC [10]	1024	64	2000	4000	64	1024	3e-6	0.3	0.2	4
ME [11]	256	8	1000	2000	32	2048	2e-5	0.2	0.3	5
MPMC [12]	256	8	1000	2000	32	2048	2e-5	0.2	0.3	5
MC [13]	256	8	1000	2000	32	2048	2e-5	0.2	0.3	5
ML [5]	256	8	1000	2000	32	2048	2e-5	0.2	0.3	5

8. Evaluation Metrics

To evaluate the performance of SpaDiT and other baseline methods, we use five evaluation metrics to evaluate the gene prediction performance of different methods on ten datasets. The specific metrics are as follows:

- **PCC**: The Pearson correlation coefficient (PCC) value was computed using the following equation:

$$\text{PCC} = \frac{E\left[(x_i - \mu_i)(\hat{x}_i - \hat{\mu}_i)\right]}{\sigma_i \hat{\sigma}_i} \tag{25}$$

  where x_i and $\hat{x}_i$ denote the spatial expression vectors of gene i in the ground truth and the predicted result, respectively; μ_i and $\hat{\mu}_i$ represent the average expression values of gene i in the ground truth and the predicted result, respectively; and σ_i and $\hat{\sigma}_i$ denote the standard deviations of the spatial expression of gene i in the ground truth and the predicted result, respectively. For a single gene, a higher Pearson correlation coefficient (PCC) value indicates superior prediction accuracy.
- **SSIM**: The Structural Similarity Index Measure (SSIM) value was computed using the following equation:

$$SSIM = \frac{(2\mu_i\mu_i' + C_1^2)(2cov(x_i, \hat{x}_i) + C_2^2)}{(\mu_i^2 + \mu_i'^2 + C_1^2)(\sigma_i^2 + \sigma_i'^2 + C_2)} \tag{26}$$

  where the definitions of μ_i, μ_i', and σ_i' are analogous to those used in calculating the Pearson correlation coefficient (PCC) value; C_1 and C_2 are 0.01 and 0.03, respectively; and $cov(x_i, \hat{x}_i)$ represents the covariance between the expression vector of the gene in the ground truth (x_i) and that in the predicted result ($\hat{x}_i$). For a single gene, a higher Structural Similarity Index Measure (SSIM) value indicates superior prediction accuracy.
- **RMSE**: We utilized the following equation to calculate the Root Mean Square Error (RMSE):

$$RMSE = \sqrt{\frac{1}{M}\sum_{j=1}^{M}(z_{ij} - \hat{z}_{ij})^2} \tag{27}$$

  where z_{ij} and $\hat{z}_{ij}$ represent the z-scores of the spatial expression of gene i in spot j in the ground truth and the predicted result, respectively. For a single gene, a lower Root Mean Square Error (RMSE) value indicates superior prediction accuracy.

- **JS**: Jensen-Shannon (JS) divergence uses relative information entropy, specifically Kullback–Leibler (KL) divergence, to quantify the difference between two distributions. Initially, we computed the spatial distribution probability of each gene as follows:

$$P_{ij} = \frac{x_{ij}}{\sum_{j=1}^{M} x_{ij}} \tag{28}$$

where x_{ij} denotes the expression of gene i in spot j, M represents the total number of spots, and P_{ij} is the distribution probability of gene i in spot j. Subsequently, we calculated the Jensen-Shannon (JS) divergence value for each gene using the following equation:

$$JS = \frac{1}{2}KL\left(P_i, \frac{P_i + \hat{P}_i}{2}\right) + \frac{1}{2}KL\left(\hat{P}_i, \frac{P_i + \hat{P}_i}{2}\right) \tag{29}$$

where P_i and $\hat{P}_i$ represent the spatial distribution probability vectors of gene i in the ground truth and the predicted result, respectively. The Kullback-Leibler (KL) divergence between two probability distributions a and b is defined as:

$$KL(a||b) = \sum_{j=0}^{M}(a_j \times \log \frac{a_j}{b_j}) \tag{30}$$

where a_j and b_j represent the defined probabilities and actual probabilities of gene i in spot j, respectively. For a single gene, a lower Jensen-Shannon (JS) divergence value indicates superior prediction accuracy.
- **AS**: We defined the Accuracy Score (AS) by aggregating the Pearson correlation coefficient (PCC), Structural Similarity Index Measure (SSIM), Root Mean Square Error (RMSE), and Jensen-Shannon (JS) divergence to evaluate the relative accuracy of the integration methods for each dataset. For each dataset, we calculated the average PCC, SSIM, RMSE, and JS of all genes predicted by each integration method. Subsequently, we sorted the PCC and SSIM values of the integration methods in ascending order to obtain $\mathbf{RANK}_{\mathrm{PCC}}$ and $\mathbf{RANK}_{\mathrm{SSIM}}$; the method with the highest PCC/SSIM value was assigned $\mathbf{RANK}_{\mathrm{PCC/SSIM}} = N$, and the method with the lowest PCC/SSIM value was assigned $\mathbf{RANK}_{\mathrm{PCC/SSIM}} = 1$. We also sorted the RMSE and JS values of the integration methods in descending order to obtain $\mathbf{RANK}_{\mathrm{RMSE}}$ and $\mathbf{RANK}_{\mathrm{JS}}$; the method with the highest RMSE/JS value was assigned $\mathbf{RANK}_{\mathrm{RMSE/JS}} = 1$, and the method with the lowest RMSE/JS value was assigned $\mathbf{RANK}_{\mathrm{RMSE/JS}} = \mathrm{N}$. Finally, we calculated the average value of $\mathbf{RANK}_{\mathrm{PCC}}$, $\mathbf{RANK}_{\mathrm{SSIM}}$, $\mathbf{RANK}_{\mathrm{RMSE}}$, and $\mathbf{RANK}_{\mathrm{JS}}$ to obtain the AS value of each integration method, as follows:

$$\mathrm{AS} = \frac{1}{4}(\mathbf{RANK}_{\mathrm{PCC}} + \mathbf{RANK}_{\mathrm{SSIM}} + \mathbf{RANK}_{\mathrm{RMSE}} + \mathbf{RANK}_{\mathrm{JS}}) \tag{31}$$

For each dataset, the method with the highest Accuracy Score (AS) demonstrated the best performance among the integration methods.

9. Baselines

We compared the performance of SpaDiT to eight baseline methods, with data processing procedures (e.g., normalization and scaling) consistent for each method.

- Tangram [14]: It is a method that can map any type of sc/snRNA-seq data, including multimodal data such as those from SHARE-seq, which can be used to reveal spatial patterns of chromatin accessibility. We refer to the guide on the Tangram GitHub repository: `https://github.com/broadinstitute/Tangram`.
- scVI [15]: It is a scalable framework for probabilistic representation and analysis of single-cell gene expression. We refer to the guide on the scVI GitHub repository: `https://github.com/YosefLab/scVI`.
- SpaGE [16]: It is a method that integrates spatial and scRNA-seq datasets to predict whole-transcriptome expressions in their spatial configuration. We refer to the guide on the SpaGE GitHub repository: `https://github.com/tabdelaal/SpaGE`.
- stPlus [17]: It is a reference-based method that leverages information in scRNA-seq data to enhance spatial transcriptomics. We refer to the guide on the stPlus GitHub repsitory: `https://github.com/xy-chen16/stPlus`.
- SpaOTsc [18]: It is a method that relies on structured optimal transfer to recover the spatial properties of scRNA-seq data by exploiting spatial measurements of a relatively small number of genes. We refer to the guide on the SpaOTsc GitHub repository: `https://github.com/zcang/SpaOTsc`.
- novoSpaRc [19]: It is a method that reconstructs tissue based on this hypothesis and optionally improves the reconstruction by including a reference map of marker genes. We refer to the guide on the SpaOTsc GitHub repository: `https://github.com/rajewsky-lab/novosparc`.
- SpatialScope [20]: It is a method to integrate scRNA-seq reference data and ST data using deep generative models. We refer to the guide on the SpatialScope GitHub repository: `https://github.com/YangLabHKUST/SpatialScope`.
- stDiff [21]: It is a method that capturing gene expression abundance relationships in scRNA-seq data through two Markov processes. We refer to the guide on the stDiff GitHub repository: `https://github.com/fdu-wangfeilab/stDiff`

10. More result of hierarchical clustering for prediction accuracy evaluation

To fully demonstrate the accuracy of the SpaDiT method in predicting spot expression, we employed hierarchical clustering to visualize the similarity between the predicted spots and the true spots labels, and compared the results with those from other benchmark methods.

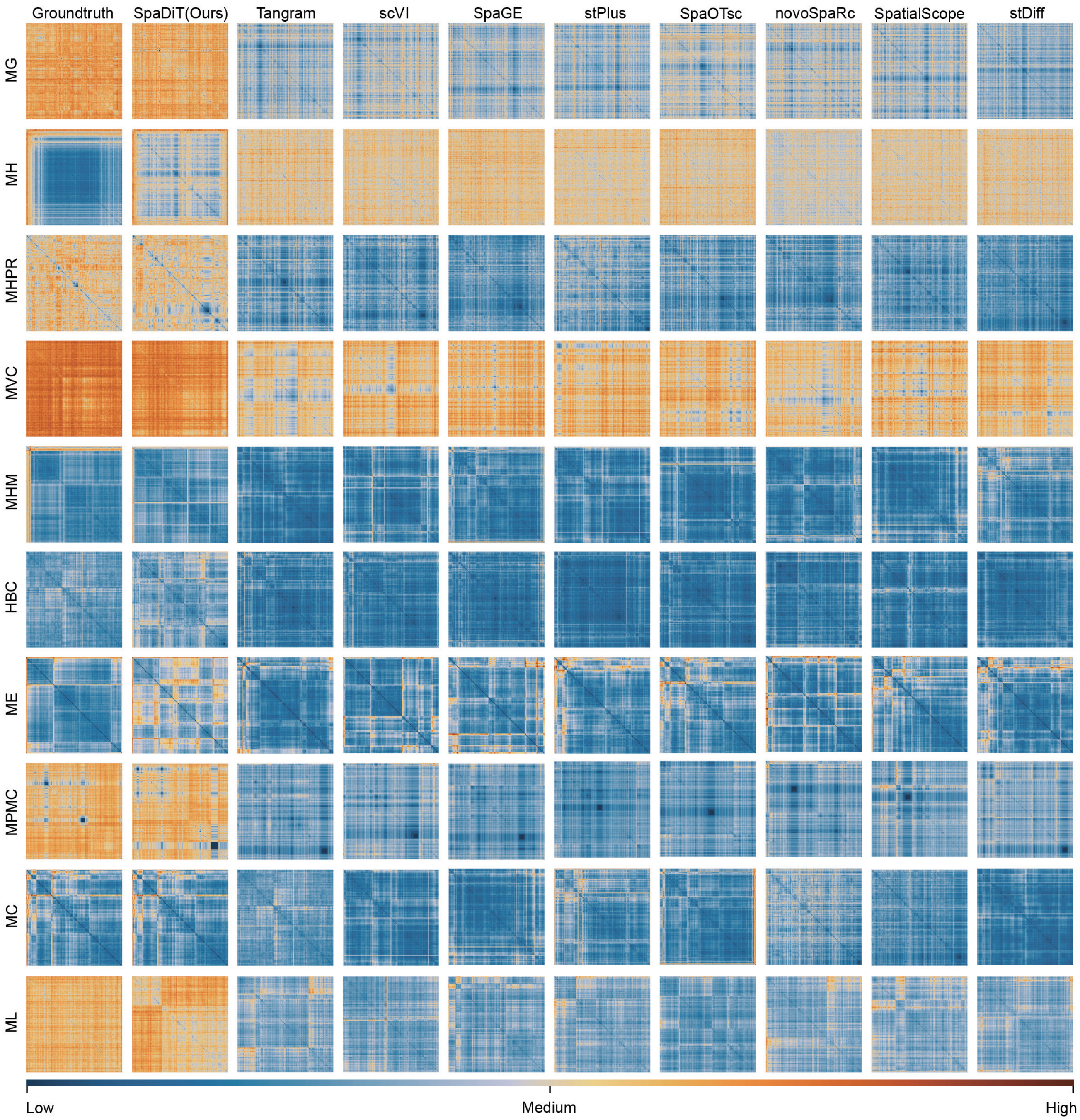

Figure S1. Visualization of the prediction performance of various baseline methods. The first column of the figure shows the results after clustering the true labels. The closer the predicted results of each method are to the true labels, the better the effect. The clustering effect of SpaDiT is closest to the true labels.

First, we calculated the Euclidean distance between each pair of spots in the spots expression matrix predicted by each method to reflect the similarity of the expression patterns of two spots: the smaller the distance, the higher the similarity. After calculating the distance of all spots pairs, we used hierarchical clustering to sort these spots to ensure that the spots within the cluster show the greatest similarity. With this sorting, we can reorganize the rows and columns of the distance matrix so that similar spots are adjacent to each other in the heat map.

As shown in the Figure S1, the first column of the figure visualizes the true spots labels after clustering. The closer the predicted spots heat map is to the true labels, the higher the prediction accuracy of the method. As evident from the figure, the prediction results of the SpaDiT method are very close to the true labels, demonstrating its high accuracy in predicting spots expression.

11. More result of visual consistency of ST spatial patterns

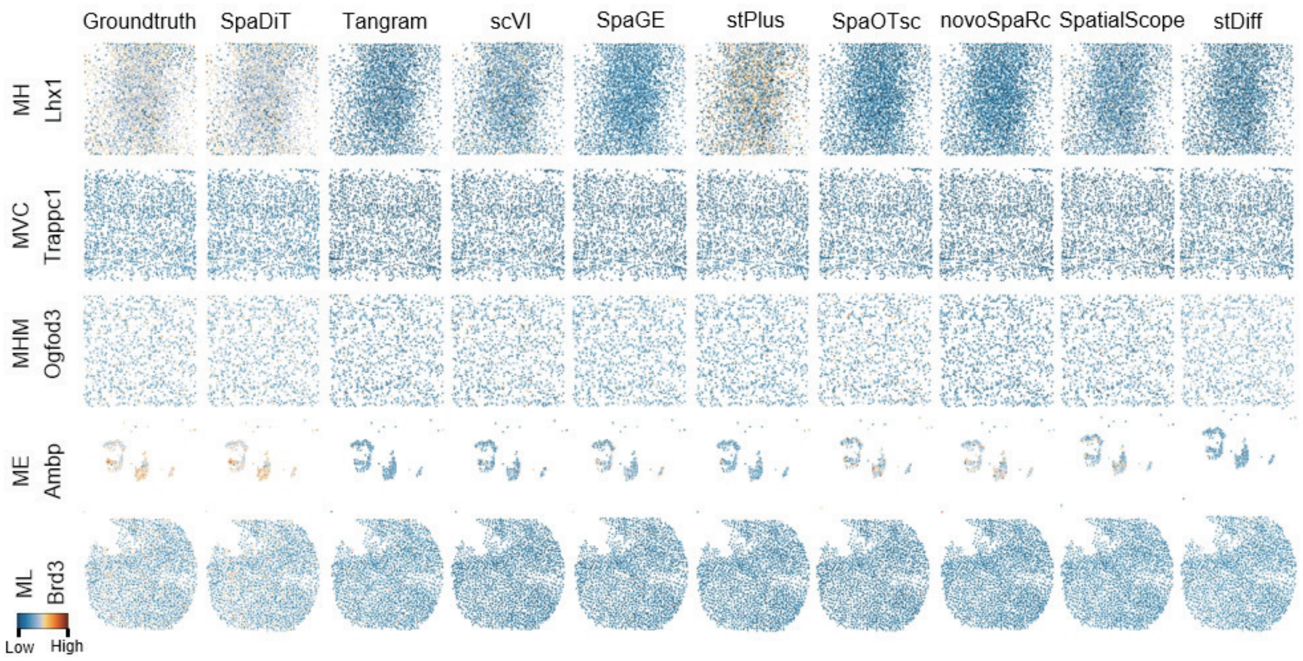

Figure S2. Predicted expression abundance of genes with known spatial patterns in four datasets. Each column corresponds to a gene with a clear spatial pattern. The first column represents the spatial pattern genes with true labels. Subsequent columns show the corresponding predicted expression patterns obtained by using SpaDiT, Tangram, scVI, SpaGE, stPlus, SpaOTsc, novoSpaRc, SpatialScope, and stDiff.

In addition to quantitatively evaluating the gene expression similarity between the true genes of ST and the genes predicted by ST, we also visually demonstrate the consistency of spatial patterns in Figure S2.

We selected five datasets with clear spatial patterns: MH, MVC, MHM, ME, and ML to illustrate the consistency of the spatial patterns between the genes predicted by the methods and the true labels. We display the predicted genes with the highest Pearson correlation coefficient (PCC) values in the datasets. T

As illustrated in Figure S2, in the ME dataset, SpaDiT restores the overall spatial pattern more accurately, followed by stDiff and stPlus, while the other methods show less obvious spatial contours in the upper right part. In the ML dataset, SpaDiT provides more accurate predictions in the middle part, while the high expression area and low expression area of other methods appear somewhat chaotic. In the MH and MVC datasets, all methods predict relatively accurate spatial patterns, but SpaDiT is the method with expression value predictions closest to the true labels.

12. More result of robustness to ST data sparsity

In order to investigate the impact of different sparsity levels on model performance (Figure S3), we examined the correlation between the performance indicators of nine methods on 10 datasets at different sampling rates and sparsity levels. The results indicate that the prediction accuracy of the algorithm is strongly correlated with the sparsity of spatial data.

13. Downstram analysis of the predicted gene expression on the MHPR dataset

To better demonstrate the significance of SpaDiT in biological discovery, we performed downstream analysis of the MHPR dataset from four perspectives: cell annotation, cell clustering, gene enrichment analysis, and identification of spatially variable genes. Since the original dataset lacks true cell type labels for each spot, we assigned the cell type with the highest proportion after Tangram [14] deconvolution as the true label. In this downstream analysis, the parameter settings for each baselines method are as follows:

- Tangram: We followed the instructions on the Tangram GitHub repository: `https://github.com/broadinstitute/Tangram`. We set the parameters as `modes = 'clusters'` and `density = 'rna_count_based'`.
- scVI: We followed the instructions on the scVI website: `https://docs.scvi-tools.org/en/0.8.0/user_guide/notebooks/gimvi_tutorial.html`. The spatial distribution of genes was obtained using the `model.get_imputed_values` function with the parameter `normalized = False`.
- SpaGE: We followed the guidelines on the GitHub repository of SpaGE: `https://github.com/tabdelaal/SpaGE/blob/master/SpaGE_Tutorial.ipynb`. If the number of genes used for integration was greater than 50 (that is, $N_{\text{gene}} > 50$), we set the parameter `n_pv` $= N_{\text{gene}}/2$.

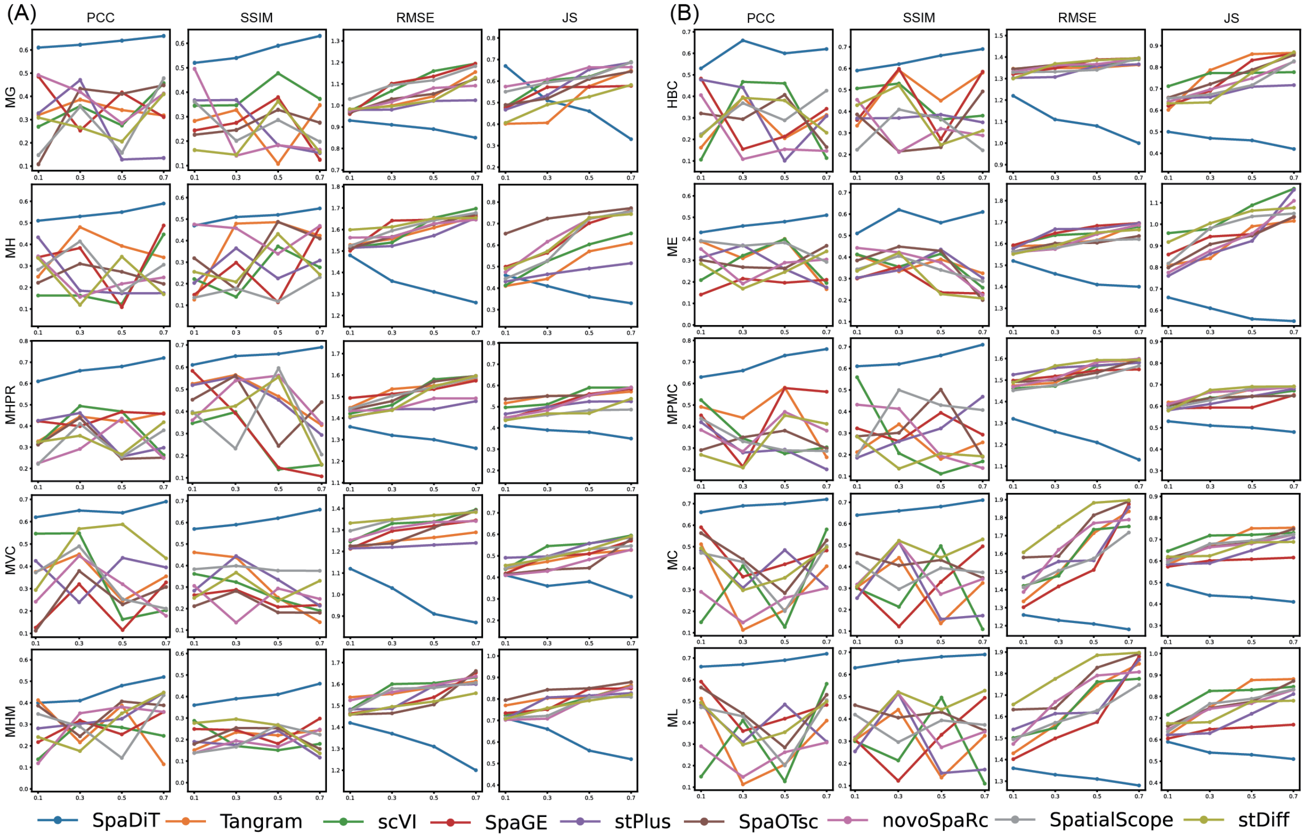

Figure S3. Comparison of model robustness at different sampling rates on four datasets (A) and four other datasets (B). We compared the changes in the four evaluation metrics of all datasets at different sampling rates. It can be seen that SpaDiT performs better than other methods at different sampling rates and has stronger model robustness.

- stPlus: We followed the instructions on the stPlus GitHub repository: `http://github.com/xy-chen16/stPlus`. We set `tmin = 5`, `neighbor = 50`.
- SpaOTsc: We followed the instructions on the SpaOTsc GitHub repository: `https://github.com/zcang/SpaOTsc`. The spatial distribution of genes was obtained using the function `issc.transport_plan` with parameters `alpha = 0`, `rho = 1.0`, `epsilon = 0.1`, `scaling = False`.
- novoSpaRc: We followed the guidelines on the GitHub repository of novoSpaRc: `https://github.com/rajewsky-lab/novosparc/blob/master/reconstruct_drosophila_embryo_tutorial.ipynb`. We set the parameters as `alpha_linear = 0.5`, `loss_fun = square_loss`, `epsilon =` 5×10^{-3}.
- SpatialScope: We followed the guidelines on the GitHub repository of SpatialScope: `https://github.com/YangLabHKUST/SpatialScope`.
- stDiff: We followed the guidelines on the GitHub repository of stDiff: `hhttps://github.com/fdu-wangfeilab/stDiff`.

We used scType [22, 23] to annotate the cells based on our predicted genes. To evaluate the accuracy of cell annotation, we used the F1-Score. As shown in Figure S4(A), we compared SpaDiT with eight baseline methods at three levels, corresponding to the colored box plots. The blue box plot represents gene expression data measured via sequencing technology, the red represents model-predicted gene expression, and the green represents the total expression data from both measured and predicted sources. Finally, to further illustrate the effectiveness of our method, we added a performance evaluation of random predictions, corresponding to the yellow section in the figure, which provides additional support for the method's effectiveness.

For cell clustering, we applied the Leiden algorithm to cluster predicted gene expression, as shown in Figure S4(B) and Figure S4(C), and used ARI to assess clustering quality.

To demonstrate the biological relevance of our predicted genes, we conducted enrichment analysis, as shown in Figure S4(D). The pathways enriched by the predicted genes were all related to the biological functions of the preoptic area of the hypothalamus. Additionally, we displayed the spatial expression patterns of several highly variable genes [24], as shown in Figure S4(E), indicating the functional heterogeneity of this region (preoptic area of the hypothalamus).

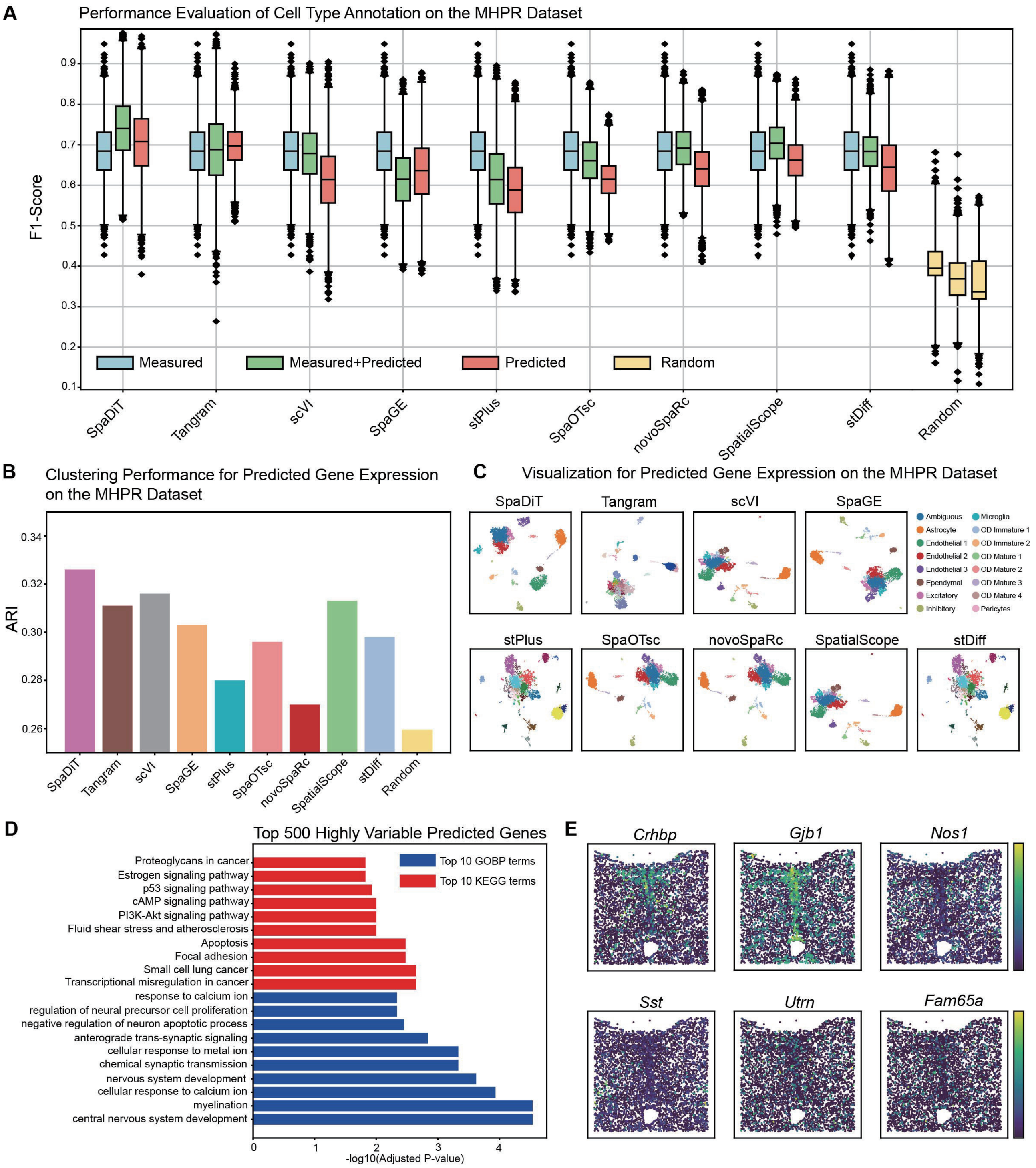

Figure S4. Overview of downstream analysis performed on the MHPR dataset. (A) Performance evaluation of cell type annotation on the MHPR dataset. The blue, green, red, and yellow box plots represent the F1 scores based on measured, combined measured and predicted, predicted, and randomly sampled gene expression (with the same number of genes), respectively. (B) Clustering performance evaluation for predicted gene expression using different methods, where the adjusted rand index (ARI) scores to assess the similarity between predicted clustering labels and true cell type labels. (C) Leiden clustering visualization, where Leiden clustering results for predicted gene expression based on the different methods. Each figure shows the cluster distribution of the same cell type in space. (D) shows top ten enrichment KEGG (red) and GOBP (blue) terms based on the top high varibale predicted genes. (E) shows the spatial distribution of the top six spatially variable genes identified by SpatialDE, and the expression of each gene is visualized in the tissue sections.

References

1. Jascha Sohl-Dickstein, Eric Weiss, et al. Deep unsupervised learning using nonequilibrium thermodynamics. In *International Conference on Machine Learning*, pages 2256–2265. PMLR, 2015.
2. Jonathan Ho, Ajay Jain, and Pieter Abbeel. Denoising diffusion probabilistic models. In *Advances in Neural Information Processing Systems*, volume 33, pages 6840–6851, 2020.
3. Jiaming Song, Chenlin Meng, and Stefano Ermon. Denoising diffusion implicit models. In *International Conference on Learning Representation*, 2020.
4. Alexander Quinn Nichol and Prafulla Dhariwal. Improved denoising diffusion probabilistic models. In *International Conference on Machine Learning*, pages 8162–8171. PMLR, 2021.
5. Nicholas Schaum, Jim Karkanias, et al. Single-cell transcriptomics of 20 mouse organs creates a tabula muris: The tabula muris consortium. *Nature*, 562(7727):367, 2018.
6. Anoushka Joglekar, Andrey Prjibelski, et al. A spatially resolved brain region-and cell type-specific isoform atlas of the postnatal mouse brain. *Nature Communications*, 12(1):463, 2021.
7. Jeffrey R Moffitt, Dhananjay Bambah-Mukku, et al. Molecular, spatial, and functional single-cell profiling of the hypothalamic preoptic region. *Science*, 362(6416):eaau5324, 2018.
8. Xiao Wang, William E Allen, et al. Three-dimensional intact-tissue sequencing of single-cell transcriptional states. *Science*, 361(6400):eaat5691, 2018.
9. David W McKellar, Lauren D Walter, et al. Large-scale integration of single-cell transcriptomic data captures transitional progenitor states in mouse skeletal muscle regeneration. *Communications Biology*, 4(1):1280, 2021.
10. Sunny Z Wu, Ghamdan Al-Eryani, et al. A single-cell and spatially resolved atlas of human breast cancers. *Nature Genetics*, 53(9):1334–1347, 2021.
11. Oraly Sanchez-Ferras, Alain Pacis, et al. A coordinated progression of progenitor cell states initiates urinary tract development. *Nature Communications*, 12(1):2627, 2021.
12. Yan Zhou, Dong Yang, et al. Single-cell rna landscape of intratumoral heterogeneity and immunosuppressive microenvironment in advanced osteosarcoma. *Nature Communications*, 11(1):6322, 2020.
13. Alla Mikheenko, Andrey D Prjibelski, et al. Sequencing of individual barcoded cdnas using pacific biosciences and oxford nanopore technologies reveals platform-specific error patterns. *Genome Research*, 32(4):726–737, 2022.
14. Tommaso Biancalani, Gabriele Scalia, et al. Deep learning and alignment of spatially resolved single-cell transcriptomes with tangram. *Nature Methods*, 18(11):1352–1362, 2021.
15. Romain Lopez, Jeffrey Regier, et al. Deep generative modeling for single-cell transcriptomics. *Nature Methods*, 15(12):1053–1058, 2018.
16. Tamim Abdelaal, Soufiane Mourragui, et al. Spage: spatial gene enhancement using scrna-seq. *Nucleic Acids Research*, 48(18):e107–e107, 2020.
17. Chen Shengquan, Zhang Boheng, et al. stplus: a reference-based method for the accurate enhancement of spatial transcriptomics. *Bioinformatics*, 37(Supplement_1):i299–i307, 2021.
18. Zixuan Cang and Qing Nie. Inferring spatial and signaling relationships between cells from single cell transcriptomic data. *Nature Communications*, 11(1):2084, 2020.
19. Noa Moriel, Enes Senel, et al. Novosparc: flexible spatial reconstruction of single-cell gene expression with optimal transport. *Nature Protocols*, 16(9):4177–4200, 2021.
20. Xiaomeng Wan, Jiashun Xiao, et al. Integrating spatial and single-cell transcriptomics data using deep generative models with spatialscope. *Nature Communications*, 14(1):7848, 2023.
21. Kongming Li, Jiahao Li, et al. stdiff: a diffusion model for imputing spatial transcriptomics through single-cell transcriptomics. *Briefings in Bioinformatics*, 25(3):bbae171, 2024.
22. Kristen Nader, Misra Tasci, Aleksandr Ianevski, Andrew Erickson, Emmy W Verschuren, Tero Aittokallio, and Mitro Miihkinen. Sctype enables fast and accurate cell type identification from spatial transcriptomics data. *Bioinformatics*, 40(7):btae426, 2024.
23. Aleksandr Ianevski, Anil K Giri, and Tero Aittokallio. Fully-automated and ultra-fast cell-type identification using specific marker combinations from single-cell transcriptomic data. *Nature communications*, 13(1):1246, 2022.
24. Valentine Svensson, Sarah A Teichmann, and Oliver Stegle. Spatialde: identification of spatially variable genes. *Nature methods*, 15(5):343–346, 2018.
